# Nano-second pulsed laser ablation of inconel 718 and MMPCD for simultaneous optimal ablation rate and surface quality

**DOI:** 10.1038/s41598-024-81233-0

**Published:** 2024-12-30

**Authors:** Ahmed Elkaseer, Islam H. Abdelgaliel, Jon Lambarri, Iban Quintana, Steffen Scholz, Mohamed F. Aly

**Affiliations:** 1 Department of Mechanical Engineering, Faculty of Engineering, The British University in Egypt (BUE), El- Sherouk City, 11837 Egypt; 2 Department of Production Engineering and Mechanical Design, Faculty of Engineering, Port Said University, Port Fuad, 42526 Egypt; 3 Department of Mechanical Engineering, School of Science and Engineering, The American University in Cairo, AUC Avenue, 11835 New Cairo, Egypt; 4 Department of Mechanical Engineering, Faculty of Engineering, Fayoum University, Fayoum, 63514 Egypt; 5 Tekniker, Basque Research and Technology Alliance (BRTA), c/Iñaki Goenaga 5, Eibar, Gipuzkoa, 20600 Spain; 6 Institute for Automation and Applied Informatics, Karlsruhe Institute of Technology, Hermann-von-Helmholtz-Platz 1, Eggenstein-Leopoldshafen, 76344 Germany

**Keywords:** Inconel 718, MMPCD, Laser fluence, Laser scan speed, Ablation rate, Surface roughness, Multi-objective optimization, Mechanical engineering, Materials science

## Abstract

This study investigates the ablation performance of Inconel 718, a nickel-based superalloy, and metal matrix polycrystalline diamond (MMPCD), a super composite, using a nano-second (ns) pulsed laser across a range of ablation conditions. Single trenches varying in energy fluence and scanning speeds were created, analyzing the experimental responses in terms of ablation rate and surface roughness. Using regression techniques, models were developed to understand these relationships. Four multi-objective optimization algorithms, weighted value grey wolf optimizer (WVGWO), multi-objective Pareto search (MOPS), multi-objective genetic algorithm (MOGA), and multi-objective sunflower optimization (MOSFO), were employed to optimize these models. Key findings include MMPCD achieving the highest ablation rates at maximum fluence and lower speeds with negligible recast, resulting in smoother surfaces, whereas Inconel 718 reached its peak rates at similar conditions but exhibited significant surface recast. This research provides valuable insights into ns-pulsed laser machining for advanced materials, emphasizing the impact of fluence and scanning speed on achieving high ablation rates and minimal surface roughness.

## Introduction

Nowadays, increasingly sophisticated products are being developed which necessitate new, advanced and robust materials with superior thermal and mechanical properties that include higher strength to weight ratio, better shock resistance, and improved resistance to wear, corrosion, creep and mechanical fatigue^1,2^. Such high-performance materials which include superalloys, super composites and ceramics are now indispensable for a wide range of engineering applications^3,4^.

Superalloys are metallic alloys that remain fully functioning at very high temperatures, typically higher than 500 °C which, in some applications, could be up to 90% of their melting temperature^5^. Nickel-based superalloys, (e.g. Inconel 718) and titanium-based alloys (e.g. Ti6Al4V) are the most widely used superalloys in industry^6–12^. In particular, Inconel alloys are widely used in aerospace^11^and aircraft engines^8^power engine components, turbine blades and marine parts^7,13^. Polycrystalline diamond (PCD) has become an indispensable material for aerospace, automobile and machining applications due to its superior hardness and high abrasive resistance under severe working conditions^14–17^.

However, such high performance materials are difficult to cut using conventional mechanical machining, due to their high strength, high work hardening and chemical affinity with tool materials^16,18–21^. Thus, it is not surprising that non-contact machining techniques, especially laser ablation, are used to machine these materials, with growing attention being paid by researchers and manufacturers to laser micro-milling with the goal of establishing the process as suitable for profitable implementation on an industrial scale^22^. Laser micro-machining, as a non-contact thermal material removal process, has demonstrated excellent potential to ablate a wide range of engineering materials irrespective of their mechanical properties^23^. The nanosecond (ns) pulsed laser is a thermal ablation process and normally results in a relatively large heat affected zone, but with melting effects, associated dross and recast materials^24^. It is industrially significant because it offers a compromise between high ablation rates and accuracy^25^. The capacity of the pulsed ablation process depends primarily on the thermal properties of the processed material and on the ablation conditions; laser power, pulse fluence (pulse energy per unit area), pulse duration, pulse repetition rate and the applied scanning speed^26–28^. An advantage of ns-pulsed laser ablation on conventional surface finishing processes is that laser ablation has a non-contact polishing that allows the creation of a remarkable surface quality^29,30^.

A study showed that a polycrystalline cubic boron nitride (PcBN) tool prepared by laser ablation at different scanning velocities when turning Inconel 718 at high speeds has a reduced flank wear by 40% rather than conventional tools, hence, increased tool life^31^. Another study used UV ns-pulsed laser to manufacture a skiving cutter of PCD. This study stated that a minimum surface roughness can be received at a positive defocusing amount of 100 μm and a scanning speed of 400 mm/s without a graphite phase transition. Furthermore, a superior PCD cutter with acceptable surface roughness can be obtained upon two-stage-machining^32^.

Meanwhile, a post surface finishing of selective laser melting Inconel 718 samples by laser ablation is carried out. Using statistical analysis, the optimal processing parameters were obtained at pulse energy 20 µJ and scanning speed of 5 mm/s to achieve the minimum surface roughness of 3.024 μm^33^. Moreover, the effect of laser surface remelting on 3D printed Inconel 718 parts surfaces was investigated. It is found that an inverse correlation between scan speeds during the remelting process and the size of the remelted zones was noticed, which was related to the cooling rates at different laser fluences, which had a significant impact on the thermal gradient within the remelted zone^34^.

Numerous researchers have investigated the importance of these process parameters on such factors as the ablation mechanism, material ablation rate, surface roughness and dimensional accuracy. Particular attention has been applied to the laser ablation of such materials such as aluminum 6082 where the highest pulse frequency and average power values achieved the highest MRR. On the other hand, the surface roughness is at its maximum values at the lower and upper bounds of pulse frequence and power due to the volatile nature of the material removal at high power values and the lack of sufficient power to remove the material at low power values^35^.Similarily, Chandan et al.^36^agreed that the balance of laser power energy is necessary in order to achieve better surface characteristics in addition to higher ablation rates. The surface roughness of ablatedTi-6Al-4 V alloy is highly affected by average laser power^37^. However, using a multi-pass processing strategy under the optimized conditions; laser power 40 W, assisted gas flow 25 L/min and 4 passes entailed the optimal conditions for laser cutting and surface texturing while using a low power laser system^38^. This is confirmed that average laser power and track overlap are the most influential parameters on the surface quality as increasing the overlapping from 50 to 80% at fixed laser power of 30 W increases the surface roughness to 2.25 times, while increasing the laser power from 30 W to 50 W in addition to 80% overlapping increases the roughness 480% approximately^39^. Additionally, the trade-off between the ablation parameters of alumina and aluminum nitride assisted in the micromachining of high intricate 3D geometries with high dimensional accuracy and surface integrity using laser fluence of 64 J/cm^2^, pulse frequency of 10 kHz and medium levels of pulse overlap^40^. Meanwhile, the laser ablation of nickel-based superalloys was investigated on bases of optimal surface quality^41,42^. Furthermore, the direct and indirect laser processing of coated and uncoated tungsten carbides (WC-Co) achieve high functional surfaces for optical structures, medical applications and tribological surfaces^43^. Eberle and Wegener^16,24^compared the picosecond (ps) and nanosecond (ns) laser ablation of WC and polycrystalline diamond metal matrix composites (MMPCD. It was found that the ns-laser ablation of MMPCD leave a graphitic carbon layer that in return create residual thermal stresses on the surface of the ablated workpiece. In order to detect and overcome this graphitisation mechanism, a focused ion beam was used, and the graphite layer was 0.5 μm. A 3D chip breaker with high surface quality and very accurate dimensions was manufactured by ns laser ablation successfully^17^. The cutting insert with ablated chip breaker showed superior performance rather than the insert without chip breaker as the chip breaker produced a curled chip with smooth surface without any elevations in the cutting force. Furthermore, a framework of controlled 3D micro-features by pulsed laser ablation technique was proposed by^26^. This model was developed based on calibration using trenches, as opposed to earlier modelling systems that use single craters, making calibration prone to error for a few pulses due to variations in crater shape and depth. This model enabled the pulsed laser machining of complex 3D shapes without the need for costly experimental trials. The manufacturing of polycrystalline diamond (PCD) cutting tools using pulsed laser systems showed significant outcomes rather than using grinding technique. The pulsed laser system has no tool cost or wear in addition to the reduced manufacturing time^44^. Odake et al.^45^ demonstrated that the nano-pulsed laser ablation is more suitable for nano-polycrystalline diamond (NPD) than the single crystal diamond (SCD) due to the formation of linear diamond-graphite interface reaching a roughness of less than 100 nm in the NPD. Meanwhile, the SCD entailed curved and sharp folding points. Most of these, and other reported studies were restricted to investigating a single variable, such as ablation rate, or dimensional accuracy or surface roughness, with few attempts to examine the effects on the laser ablation process of simultaneous changes in more than one variable. The authors have found that reports the literature of studies that investigated simultaneous optimization of laser process parameters to achieve the best ablation response are rare.

To enable successful laser machining of high-performance materials and to advance ns-pulsed laser technology, it is important to optimize the process. This research work aims to help fulfil that objective, in particular by optimizing ns-pulsed laser machining to enable controllable and predictable performance of laser milling when processing advanced materials. This study carried out an experimental investigation by ablating single trenches in Inconel 718 and MMPCD, under a wide range of ablation conditions. Ablation performance was evaluated with regard to resulting ablation rate and obtained surface roughness at the center line of the generated trenches. Then, regression modelling was used to establish non-linear relationships between experimental input parameters (scanning speed and pulse fluence) and measured output parameters (surface roughness and ablation rate). Then, the models were processed to identify the best ablation parameters for highest possible ablation rate while maintaining the surface roughness as low as possible.

The remainder of this paper is organized as follows. The experimental work is presented and the results obtained analyzed. Next the implementation of the regression modelling is introduced and this is followed by the employment of a number of multi-objectives optimization techniques to identify the optimal process parameters. Next, the experimental validation is discussed. Finally, conclusions are drawn and possible future research perspectives are outlined.

## Experimental work

### Workpiece material

In this study, two different advanced materials were used for the experimental trials; Inconel 718 and MMPCD, the latter sample had a mirror-like surface, but this was not the case for the other Inconel 718 sample. Thus, prior to the ablation tests, both tops and bottoms of the sample were prepared to be almost perfectly parallel to avoid any variation of sample height over the scanning area. Additionally, the top surfaces of the sample were polished to a mirror finish to eliminate any uncertainties in the laser ablation process associated with topography and height variation of the workpiece surface.

Table 1 shows the chemical compositions of the nickel and titanium alloys. These were obtained using Energy Dispersive X-ray Spectroscopy and a Scanning Electron Microscope.

**Table 1:** Chemical compositions of nickel alloy (Inconel 718).

**Element**	**Ti**	**Cr**	**Ni**	**Nb**	**Mo**	**Fe**
**%**	0.35	19.4	53.1	5.4	3.2	Balance

### Ns-laser ablation

A ns-pulsed laser, with a wavelength of 1064 nm and a maximum (average) power of 40 W was used for the ablation tests. The laser, an ytterbium-doped fibre laser, capable of producing pulses at variable repetition rates and durations, see Table 2, was manufactured by SPI.

**Table 2:** Ablation conditions for the initial trials.

**Waveform Number**	**Emax (mJ)**	**Pulse Duration (ns)**	**PRF (kHz)**	**Fluence (J/cm2)**	**Average Energy (W)**
0	1.33	250	30	85.33	40
14	1.05	200	38	67.37	40
1	0.85	130	47	54.54	40
2	0.53	60	76	34.00	40
25	0.37	40	108	23.74	40
3	0.28	30	145	17.96	40
5	0.16	9	250	10.27	40

**Fig. 1:**
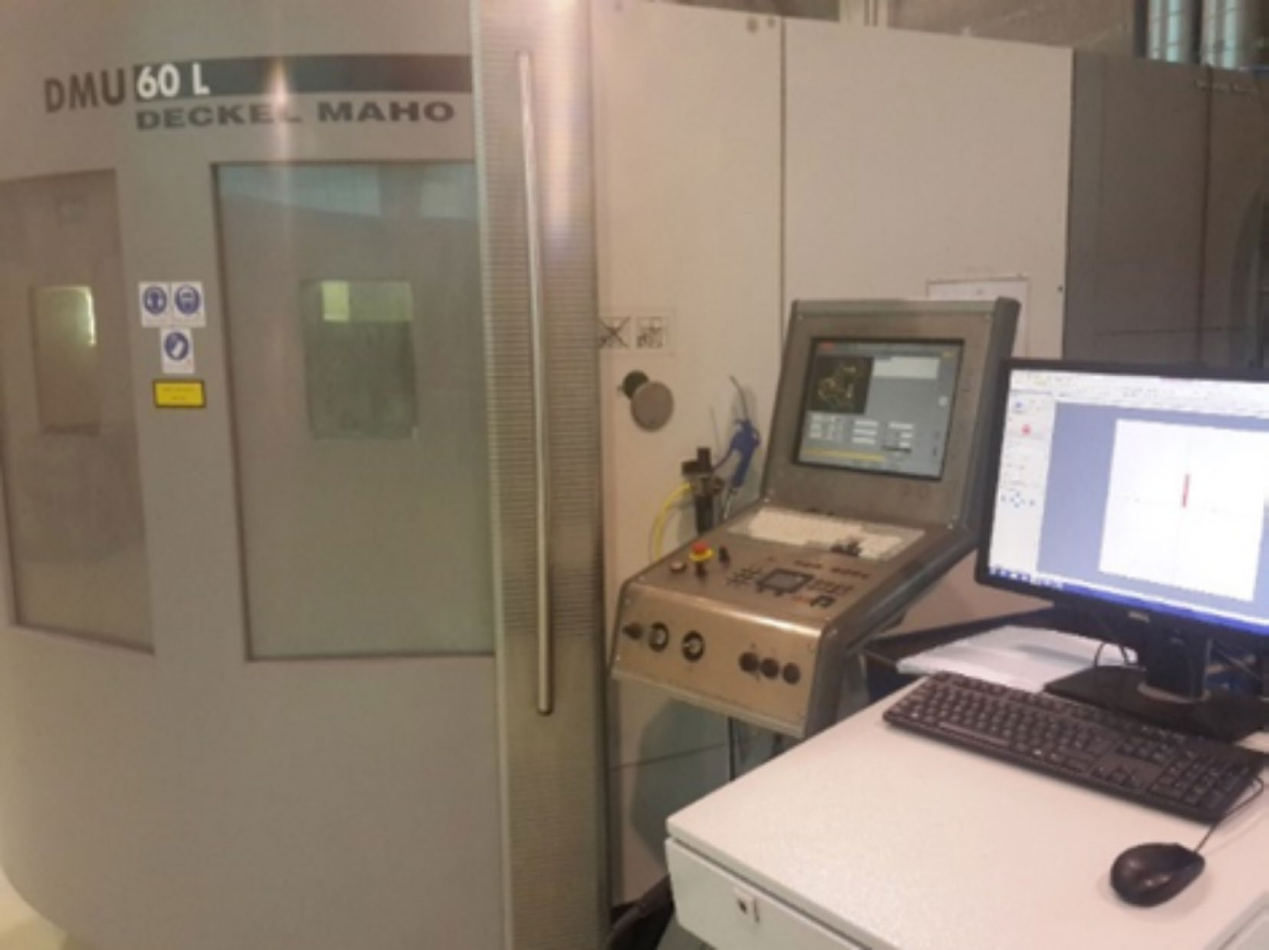
Laser work station.

To allow the workpiece to be accurately positioned with respect to the beam, the laser system was mounted on a Deckle Maho 3-axes milling machine, see Fig. 1. The laser was equipped with a 3-D galvo scanning unit for precision scanning, a collimating unit of 30 mm focal length to minimise dispersion, and a 100 mm F-theta focusing unit to minimise distortion.

**Fig. 2:**
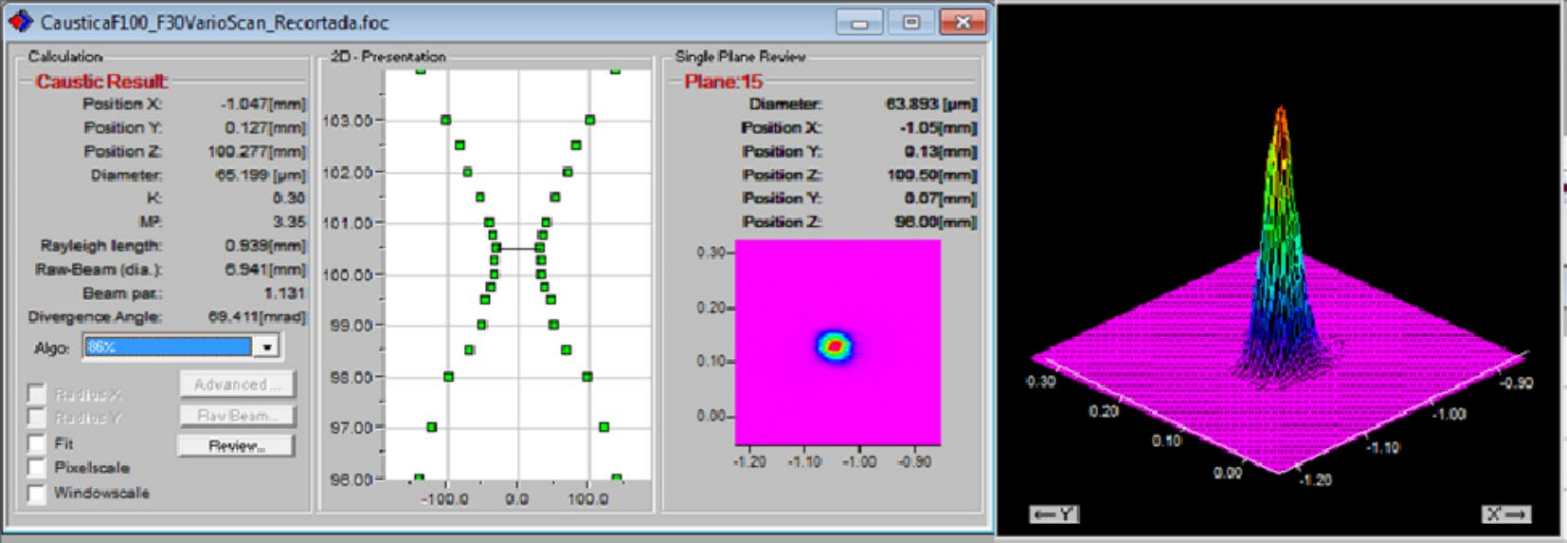
Characterization of the laser beam of SPI-G4-HS ns ytterbium-doped fiber laser.

The laser emitted a pulse with a profile in a plane perpendicular to the beam axis that was Gaussian, see Fig. 2. The laser beam caustic was characterised experimentally by a mechanical scanning diagnostic system (FocusMonitor, Primes). A laser beam quality, M^2^ ≈ 3.35, was identified with the following propagation parameters: Rayleigh length Z_R_=650 μm and beam waist (minimum spot radius ω) of 31.5 μm, see Fig. 2.

### Ablation conditions

**Fig. 3:**
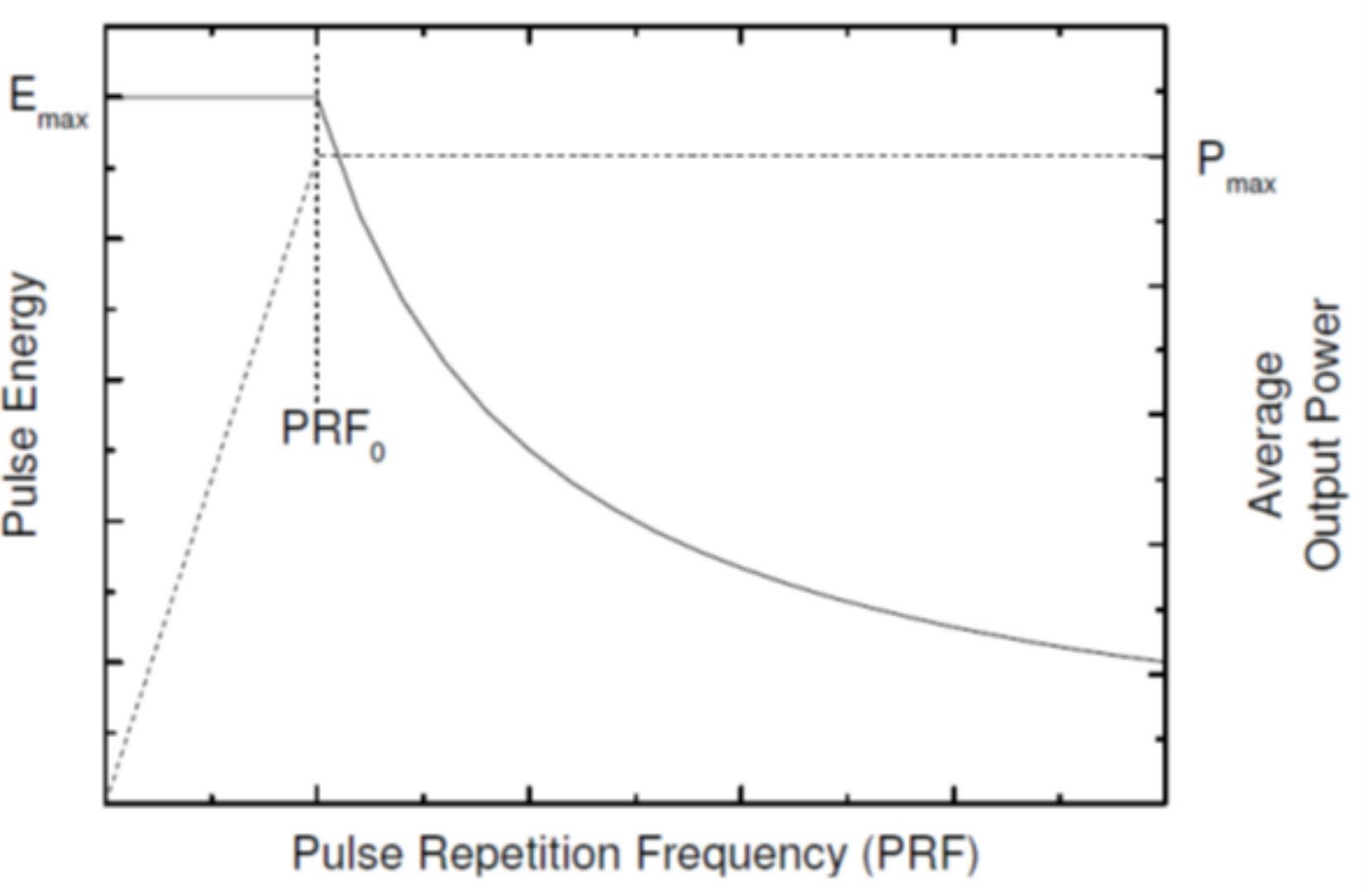
Average power and pulse energy vs. pulse repetition frequency^46^.

The pre-programmed waveform facility, available in the SPI laser source, was used to control the emitted laser pulses during the experimental trials in this study. Each waveform had a specific pulse shape and duration, and the frequency was tunable to optimize the energy and power of the delivered laser pulses, as shown in Fig. 3^46^.

For each waveform there is a threshold frequency called “PRF0”, below this frequency the average power is reduced in order not to damage the laser. When a value higher than the PRF0 is set, the average power is maintained at the maximum level while the energy per pulse decreases as the frequency increases. Setting the threshold frequency optimises the pulse, so that the energy per pulse is delivered at the maximum average power. As an example, Waveform “0” applies the maximum peak energy available in the laser system, Emax = 1.33 mJ per pulse, with a pulse duration of 250 ns and corresponding optimal pulse repetition rate (PRF) is 30 kHz, to deliver the maximum power of 40 W. Waveform “5” denotes the minimum peak energy of 0.16 mJ per pulse, with a pulse duration of 9 ns and pulse repetition rate of 250 kHz to ensure the maximum power of 40 W. Setting the optimal value of pulse repetition rate for each waveform maintains the average power at the maximum level, 40 W.

In this study, a wide range of available waveforms was applied, as shown in Table 2. This range of peak pulse energies was from 1.33 mJ to 0.16 mJ, with corresponding pulse durations from 250 ns to 9 ns, and corresponding fluences from 85.33 J/cm^2^ to 10.27 J/cm^2^. For each waveform the appropriate PRF was used so the average power was maintained at the maximum value, 40 W.

For each of the seven waveforms, the scanning speeds were varied between 0.1 m/s and 1.0 m/s. Because of a single, simple trench is the most basic cut in 3D machining, this investigation of ablation performance was limited to producing simple trenches of length 1 mm for every combination of ablation parameters and conditions. This allowed identification and assessment of the effects of the different process conditions (fluence and scanning speed), on the ablation rate and achieved surface roughness along the centre of the ablated trenches.

In this study, it is crucial to understand the inverse relationship between scanning speed and fluence, which significantly affects the ablation outcomes. Fluence is defined as the laser energy delivered per unit area, calculated as the product of pulse energy and the pulse repetition frequency (PRF), divided by the product of scanning speed and beam spot size. Consequently, when the pulse energy and PRF are held constant, a decrease in scanning speed results in an increase in the energy delivered per unit area, thereby increasing the fluence. For instance, reducing the scanning speed by a factor of ten, under constant pulse energy and PRF, results in a tenfold increase in fluence. This increased energy concentration at lower scanning speeds enhances the material removal rate, but can also lead to greater thermal effects on the material, such as a larger heat-affected zone or changes in microstructural properties. This relationship is pivotal for optimizing process parameters to balance ablation efficiency with surface integrity.

### Mathematical model development

Using MATLAB regression toolbox, the mathematical models of ablation rate and surface roughness of both materials were developed. In order to develop accurate models, the input parameters were normalized between − 1 and 1, hence, the parameters are given the subscript n. The normalization of input parameters follows (1).

1 $$\:{x}_{n}=2\frac{{x}_{i}-{x}_{min}}{{x}_{max}-{x}_{min}}-1$$

It was noticed that MMPCD material has a non-zero values in all fluence and scanning speed ranges. Meanwhile, the Inconel 718 material has only a useful range for fluence between 54.54 and 85.33 J/cm^2^ and scanning speed between 200 and 1000 mm/s. The actual and normalized ablation conditions of both materials are presented in Table 3.

**Table 3:** Ablation conditions for the experimental trials.

Variable	Actual values	Normalized values in (1)
Influence	MMPCD $$\:\left[\begin{array}{ccccc}23.74&\:34&\:54.54&\:67.37&\:85.33\end{array}\right]$$ Inconel 718 $$\:\left[\begin{array}{ccc}54.54&\:67.37&\:85.33\end{array}\right]$$	MMPCD $$\:\left[\begin{array}{ccccc}-1&\:-0.66&\:0&\:0.416&\:1\end{array}\right]$$ Inconel 718 $$\:\left[\begin{array}{ccc}-1&\:-0.17&\:1\end{array}\right]$$
Scanning speed	MMPCD $$\:\left[\begin{array}{cccc}100&\:200&\:\dots\:&\:1000\end{array}\right]$$ Inconel 718 $$\:\left[\begin{array}{cccc}200&\:300&\:\dots\:&\:1000\end{array}\right]$$	MMPCD $$\:\left[\begin{array}{cccc}-1&\:-0.778&\:\dots\:&\:1\end{array}\right]$$ Inconel 718 $$\:\left[\begin{array}{cccc}-1&\:-0.75&\:\dots\:&\:1\end{array}\right]$$

### Multi-objective optimization algorithms

Four multi-objective optimization algorithms are used; (1) weighted value grey wolf optimizer (WVGWO)^47,48^, (2) multi-objective pareto search (MOPS) (3) multi-objective genetic algorithm (MOGA) and (4) multi-objective sunflower optimization (MOSFO)^49^. The first three algorithms showed great performance in similar problems but different manufacturing processes^50,51^. However, a new algorithm MOSFO is selected alongside the tested algorithms as the developer claimed that it outperformed ten benchmarked algorithms.

**Fig. 4:**
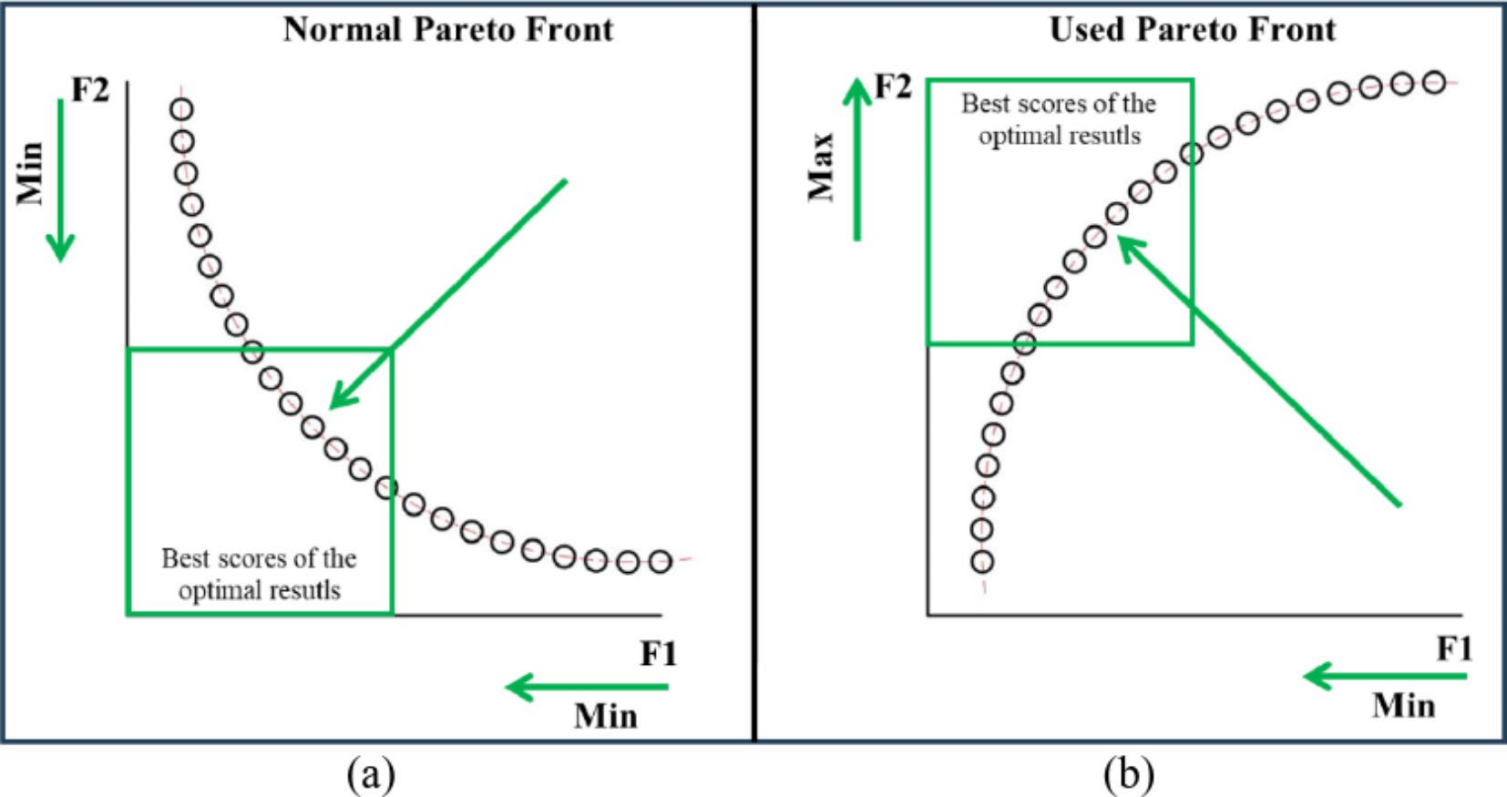
Simple illustration of the difference between (**a**) normal and (**b**) flipped pareto front.

The main objective functions of the multi-objective optimization model are the minimization the surface roughness and the maximization of the ablation rate. Generally, almost all algorithms are developed to find the global minima of the given objective, hence, the second objective can be replaced by the minimization of the additive inverse of the ablation rate. The objective functions are (2) and (3). This reflects on the pareto front plot to be reversed as the results of the negative objective in (3) is returned to its original positive value. The normal two objectives plot on pareto front and the used pareto front are illustrated in Fig. 4.

2 $$\:Min\left(Ra\right)$$

3 $$\:Min\left(-MRR\right)$$

## Results and discussion

**Fig. 5:**
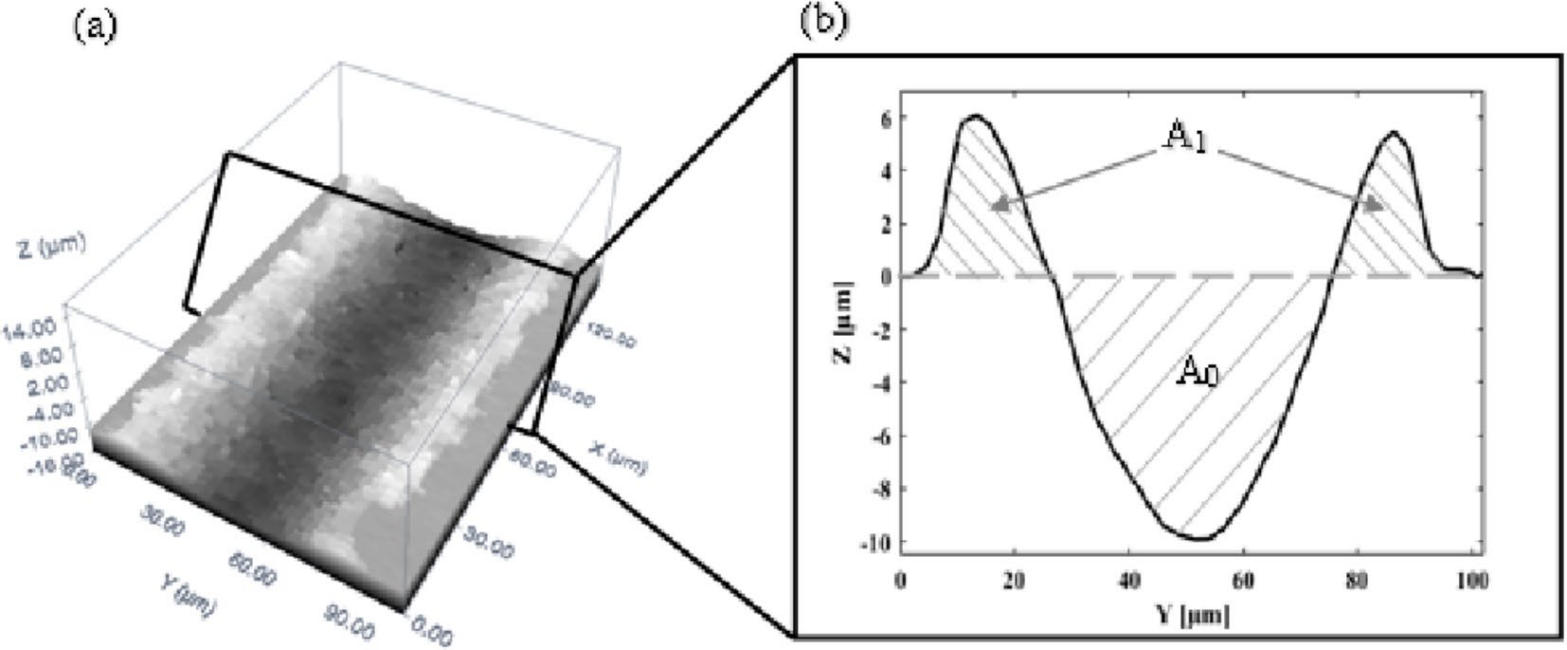
Example of (**a**) 3D image of trench, and (**b**) cross-section of the ablated trench.

A Nikon ME600, confocal microscope with 100X objective lens was used to assess the quality of the ablated grooves. To avoid the influence of any dynamic instability of the laser system, such as acceleration and deceleration of the laser scanner at the start and end of its scan, only the middle area of each trench was captured for further processing and analysis. In particular, an area of 138.88*102.02 µm^2^ (equivalent to 762*560 pixels) was scanned, see Fig. 5a. The captured images were converted into *.dat files using the confocal optical imaging profiler (Plu Ver. 1.7). These files were read by a MATLAB script reader developed by the authors to extract the average profile of the measured trench, see Fig. 5b.

It is well known that with the ns-pulsed laser, material removal is by thermal ablation. The laser pulses deliver energy in the form of heat into the target material’s surface where, as a result, a proportion of the irradiated material is vaporised while the surrounding area is melted. Vapour pressure due to the laser generated evaporation expels the molten material from the interaction zone. Some of this material solidifies and forms droplets and recast burrs. Thermal processes such as this can have negative effects, including generating micro-cracks, deterioration of the microstructure, morphology changes which reduce obtainable surface quality, and pile-up of the recast.

Figure 5 shows a typical result when ablating material using a ns-pulsed laser. Here A_0_ is the cross-section of the ablated material which is removed, and which typically has the form of a U-shaped trench. A_1_ is the cross-sectional area of the piled-up material due to recast. Here the ablation rate was found in two ways. In the first, it was equated to A_0_ as shown in Fig. 5, the material removed from the trench by evaporation and melt expulsion, ignoring the recast material. This is dubbed the “nominal material removal rate”. In the second method, the recast material is subtracted from the nominally removed material (A_0_ - A_1_). This is the “effective material removal rate” and is the ablated material permanently removed from the workpiece.

The “nominal material removal rate” can be useful when studying the patterning of the surface, when the recast material is considered as part of the patterning. The “effective material removal rate” is useful when, for example, applying laser milling, and the rate at which material is completely and actually removed is important.

As stated above, a MATLAB sub-routine was composed to read the captured 3D data for the trenches produced and determine the average profiles of A_0_ and (A_0_-A_1_). The nominal and effective material removal (ablation) rates were then found by multiplying the scanning speed by the area A_0_ or (A_0_-A_1_), respectively.

**Fig. 6:**
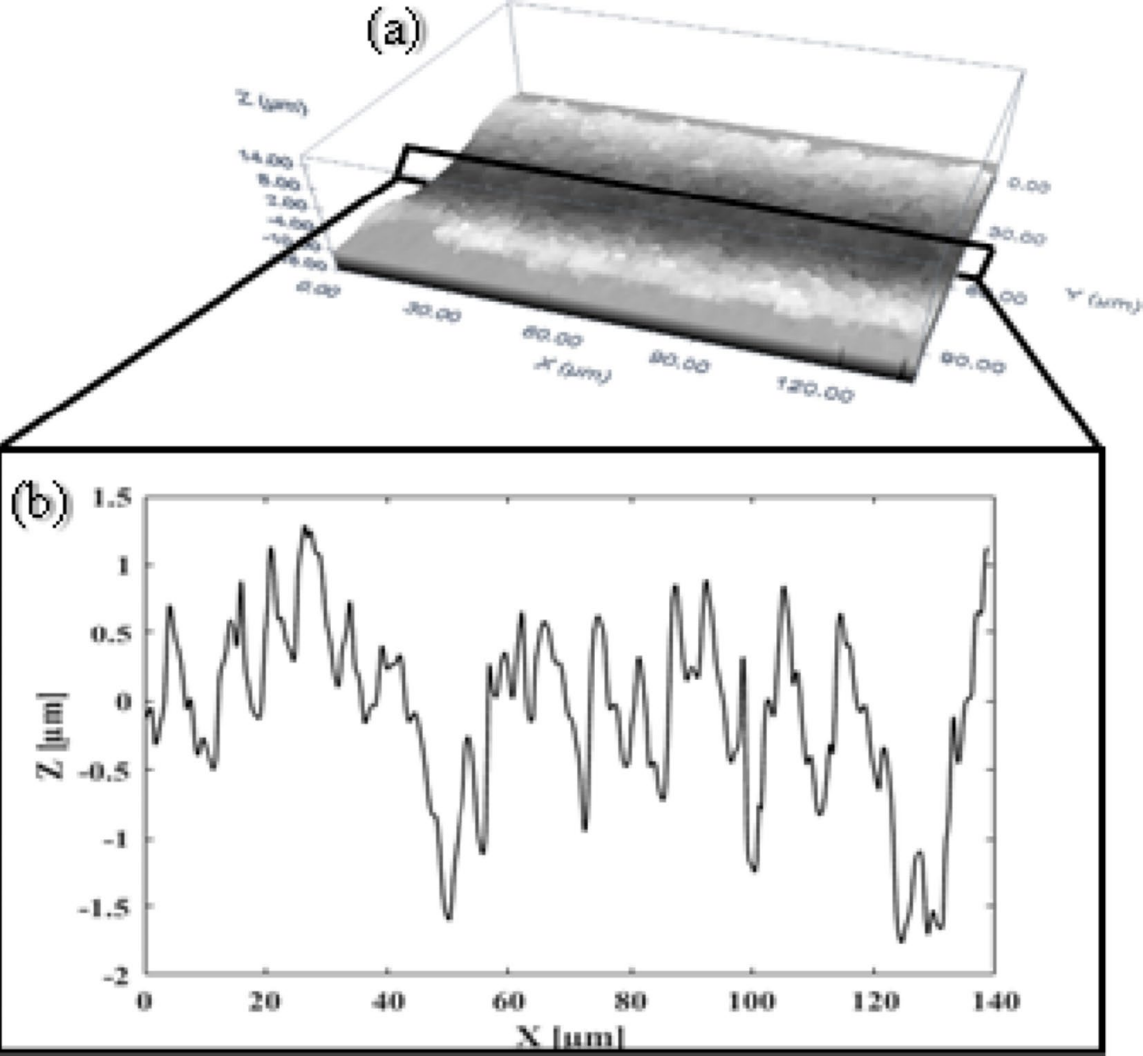
An example of measured roughness along the centre line of a trench (**a**) 3D image and (**b**) 2D image of the measured surface roughness.

Figure 6 shows the measured roughness along the centre line of the ablated trench, found by applying the Plu Ver. 1.7 confocal optical imaging profiler to the extracted data. R_a_ is the averaged surface roughness.

### Ablation rate experimental results

**Fig. 7:**
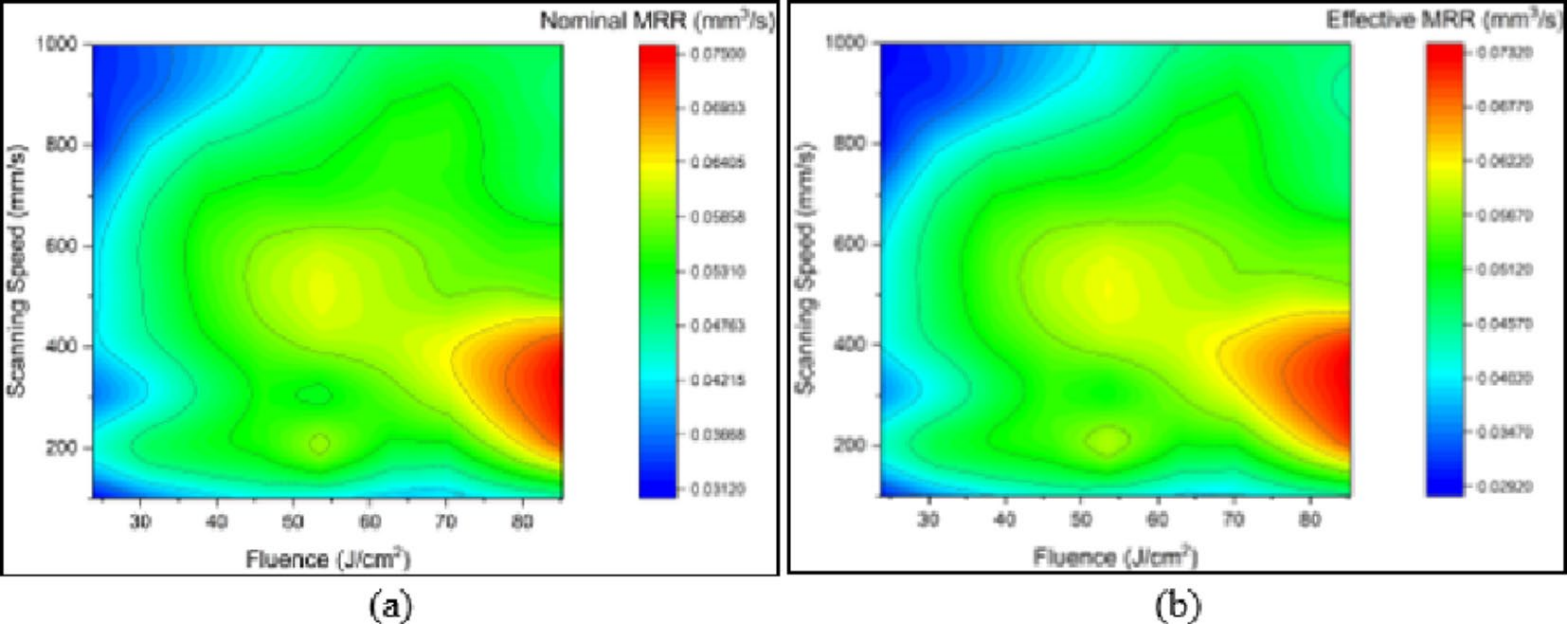
Effect of fluence (J/cm^2^) and scanning speed (mm/s) on (a) nominal ablation rate (mm^3^/s) and (b) effective ablation rate (mm^3^/s), for MMPCD.

**Fig. 8:**
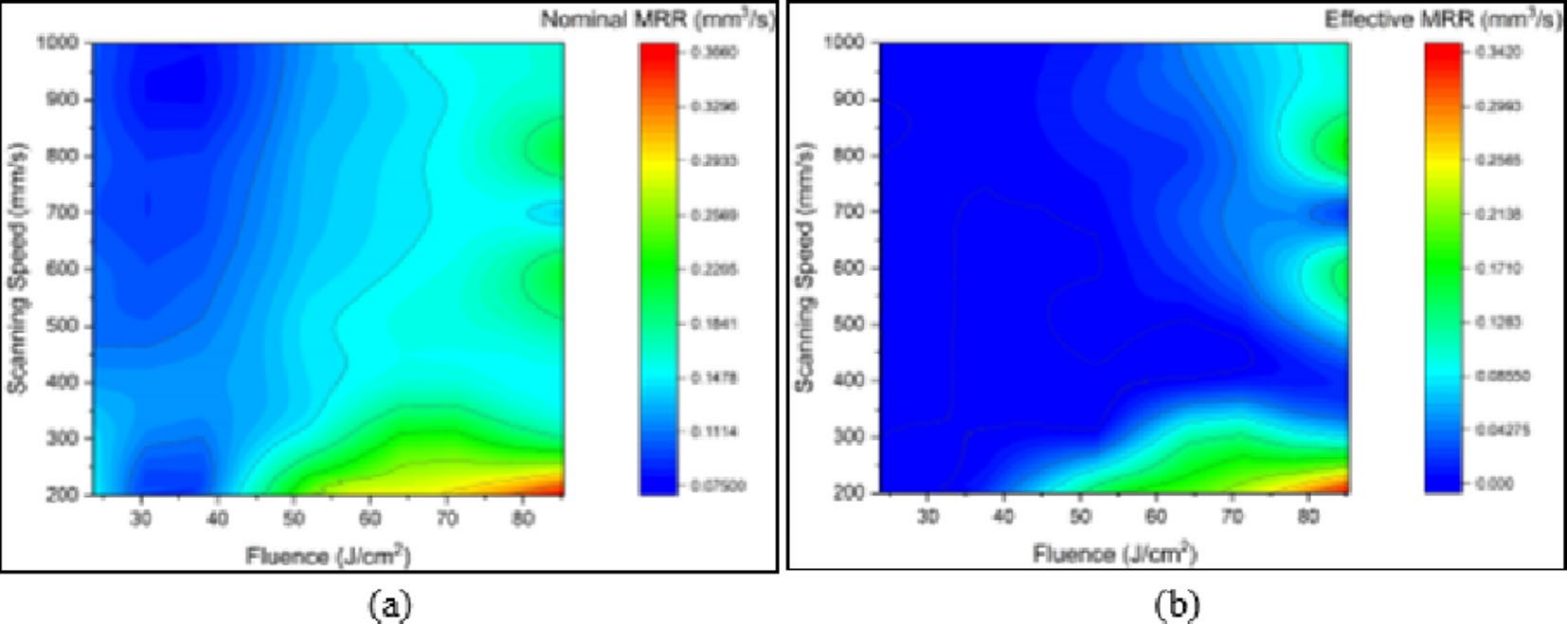
Effect of fluence (J/cm^2^) and scanning speed (mm/s) on (a) nominal ablation rate (mm^3^/s) and (b) effective ablation rate (mm^3^/s), for Inconel 718.

Figures 7 and 8 are 2D planar maps representing nominal and effective ablation rates for each of the three materials used. Each figure is for a different test material, and in each figure one panel represents effect of changes in fluence and the other in scanning speed.

Fluence levels of 10.27 J/cm^2^ and 17.96 J/cm^2^ were below the threshold for ablation and have not been included in the results presented.

Figure 7shows the nominal and effective ablation rates for MMPCD. It can be seen that the results for both cases are similar, with few observed differences. This is because when ablating MMPCD most of the material is evaporated, and only a marginal amount of material is melted and relocated^15–17^. The highest ablation rates (0.078 mm^3^/s) were obtained at the highest fluence (85.33 J/cm^2^) and relatively low scanning speeds, ranging between 200 mm/s and 400 mm/s. Minimum ablation rates (less than 0.035 mm^3^/s) were found at the minimum fluence (23.74 J/cm^2^) and at very high and, surprisingly, very low scanning speeds. These results confirm that ablation rates in MMPCD are highly dependent on fluence.

For Inconel 718, see Fig. 8, the maximum nominal and effective ablation rates were, respectively, 0.36 mm^3^/s and 0.34 mm^3^/s, and were again obtained in the region of maximum fluence (85 J/cm^2^) and low scanning speed (200 mm/s). Low nominal ablation rates were obtained at low fluence and medium to high scanning speeds. Low effective ablation rates were obtained at low fluence for the whole range of scanning speeds. It is not difficult to see noticeable differences between Fig. 8a and b which indicates that a large proportion of ablated material removed by melt expulsion was recast, especially at low values of fluence. Also, it is worth mentioning that, at the lowest scanning speed of 100 mm/s, no proper trenches (U-shaped trench) were obtained for the entire range of fluence. This could be attributed to the effect on the ablation process due to the relatively low thermal conductivity of Inconel 718 compared to MMPCD, which resulted in steep temperature gradients. The energy from the laser, even at very low scanning speeds, raised the surface temperature of the alloy and melted it, more than ablating it. The result was the formation of a large amount of debris in the target area which detracted from the geometry of the ablated trench. However, Fig. 8, shows that for Inconel 718, fluence is a more effective variable than scanning speed when determining ablation rate.

As with Inconel 718, no useful trenches were generated at the very low scanning speed of 100 mm/s, and again this was attributed to the relatively low thermal conductivity of Inconel 718 (with respect to MMPCD) so that a large proportion of the debris was not ejected out of the ablated area. The clear differences between Fig. 8a and b again demonstrate the significant contribution of the thermal conductivity effect when ablating Inconel 718 at ns-pulse duration, where ablated material removed via melt expulsion was redeposited as debris. It is worth emphasizing that, considering the conditions under which the maximum and minimum ablation rates were obtained, Inconel 718 demonstrated a response where the ablation rates were more affected by fluence than scanning speed.

Comparing the maximum ablation rates of the two materials at their optimum conditions, Inconel 718 showed ablation rates that were much higher than for MMPCD. The results for Inconel 718 revealed that ablation rates are very much influenced by thermal factors which can result in large amounts of recast material, where MMPCD came second but with almost no redeposited materials detected. Generally, it was found that fluence had a more significant effect on the ablation rate than scanning speed.

In this analysis of the laser ablation process, significant attention has to be given to the role of temperature gradients and the rest time between laser pulses, both of which critically influence the material removal rate (MMR). Temperature gradients, induced by the localized heating and cooling during laser pulse interactions, generate thermal stresses within the material. These stresses can alter the mechanical properties of the material temporarily, affecting its ablation resistance. A steep temperature gradient, for instance, can lead to higher thermal stress, potentially increasing micro-cracking or phase changes in the material, thereby affecting the MMR.

Furthermore, the rest time between pulses, often overlooked, plays a pivotal role in controlling the surface temperature during the ablation process. Shorter rest times may not allow sufficient cooling of the material between pulses, leading to accumulated heat within the target zone. This accumulation can elevate the baseline temperature of the material for subsequent pulses, potentially leading to a higher-than-expected ablation rate due to reduced surface hardness and increased thermal degradation. Conversely, longer rest times allow for more effective cooling, reducing thermal effects and potentially leading to more consistent and predictable ablation results.

By incorporating these dynamics into our process analysis, it will enable to better predict and control the outcomes of laser ablation. Adjusting the pulse frequency and rest intervals could thus be utilized as a strategy to manipulate the thermal conditions during ablation, tailoring the process to specific material properties and desired outcomes.

**Fig. 9:**
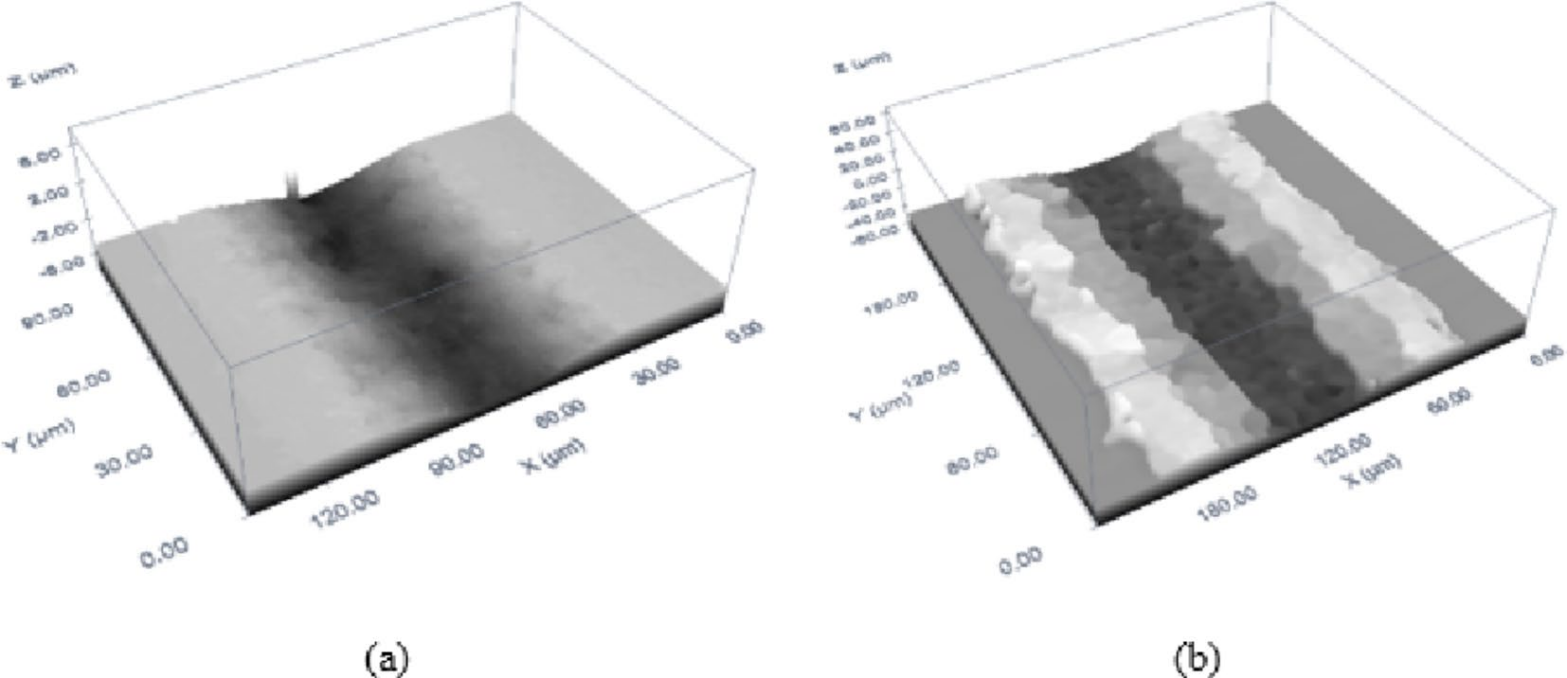
3D confocal microscopy image of an ablated trench for the optimal ablation rate of (a) MMPCD and (b) Inconel 718.

To sum up, MMPCD showed consistent ablation behaviour with the highest rates achieved at high fluence and low scanning speeds, demonstrating that ablation primarily occurs through evaporation with minimal material relocation. In contrast, Inconel 718 exhibited its highest ablation rates at similar fluence levels but was more significantly affected by the thermal properties of the material, which led to higher recast due to melt expulsion. These findings highlight the critical role of fluence in achieving high ablation rates for both materials, although the thermal behaviour of Inconel 718 introduces additional complexity in optimizing process parameters due to its propensity for generating recast material. This comparative analysis highlights the distinct responses of these materials to ns-pulsed laser ablation and the need for parameter optimization based on the specific material characteristics and desired outcomes. Finally, 3D confocal microscopy image of an ablated trench of both materials at the highest ablation rate is depicted in Fig. 9. The ablation conditions for MMPCD material in Fig. 9a are fluence of 85.33 J/cm^2^ and scanning speed of 289 mm/s resulting in a maximum ablation rate of 0.074 mm^3^/s. In case of ablated Inconel 718, Fig. 9b is captured at ablation conditions of 84.72 J/cm^2^ and 200 mm/s for fluence and scanning speed, respectively, corresponding to ablation rate of 0.341 mm^3^/s.

**Fig. 10:**
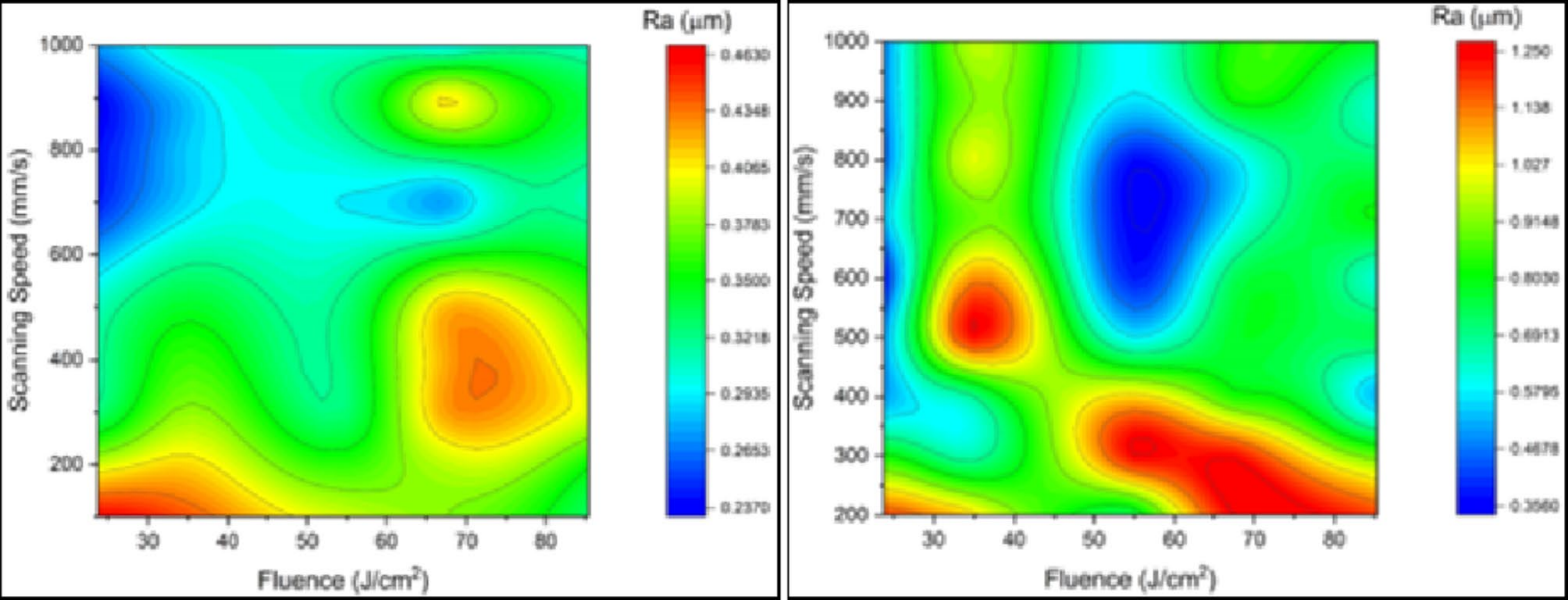
Centreline surface roughness of (a) MMPCD, (b) Inconel 718.

Figure 10a illustrates the surface roughness along the centreline of the trench generated in MMPCD. The minimum surface roughness (Ra of 0.24 μm) was observed at a low fluence of 23.73 J/cm² and high scanning speeds ranging between 700 and 950 mm/s. Conversely, the maximum roughness (0.46 μm) occurred at the same fluence but at lower scanning speeds of 100 to 150 mm/s. This disparity highlights the significant impact of scanning speed on surface roughness, suggesting that higher speeds promote smoother surfaces due to reduced heat accumulation and faster cooling, characteristics inherent to the thermal conductivity and high melting point of the cobalt matrix in MMPCD.

Figure 10b presents the surface roughness results for Inconel 718. The lowest roughness (Ra = 0.38 μm) was achieved at a medium fluence of 54.54 J/cm² and scanning speeds between 600 and 850 mm/s. The highest roughness (1.35 μm) was noted under both higher fluence (> 60 J/cm²) at scanning speeds of 200 to 300 mm/s and at a lower fluence of 34 J/cm² with medium speeds of 500 to 600 mm/s. The increased roughness in Inconel 718 at lower speeds and varying fluence levels can be attributed to the alloy’s lower thermal conductivity and higher thermal expansion, which exacerbate melt expulsion and material redeposition, leading to rougher surfaces.

**Fig. 11:**
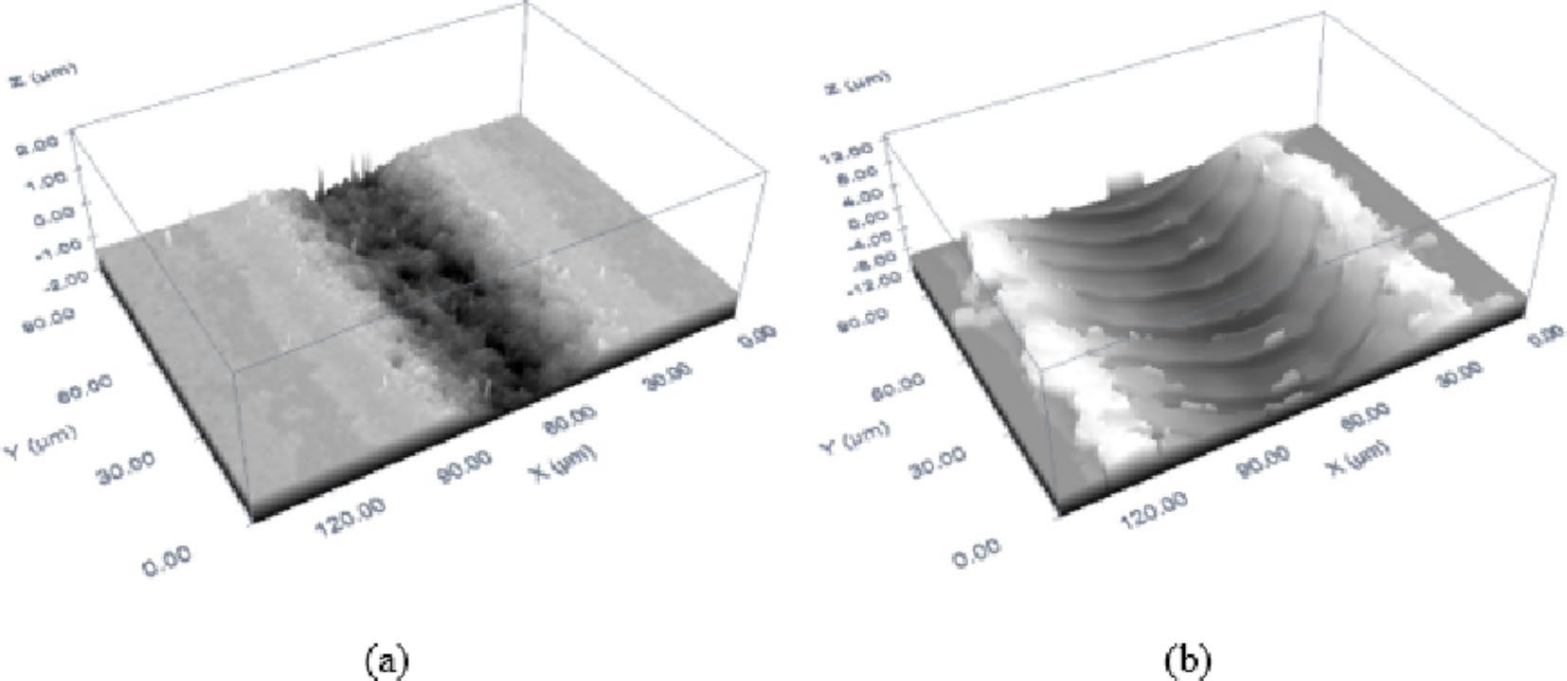
3D confocal microscopy image of an ablated trench for the optimal surface roughness of (**a**) MMPCD and (**b**) Inconel 718.

The comparative analysis of MMPCD and Inconel 718 reveals that MMPCD achieves consistently lower surface roughness, which correlates with the material’s ability to rapidly dissipate heat due to its high thermal conductivity and the stability of its diamond-cobalt matrix at high temperatures. In contrast, Inconel 718’s tendency towards higher roughness can be linked to its substantial melt expulsion, influenced by its material composition and thermal properties. High-resolution 3D confocal microscopic images have been added to visually demonstrate these differences in surface morphology post-ablation, providing a clear qualitative assessment that supports the quantitative data as shown in Fig. 11.

In summary, the investigation into surface roughness across different materials under varying laser ablation parameters revealed distinct influences of fluence and scanning speed. MMPCD consistently demonstrated lower surface roughness under high scanning speeds at low fluence, indicating that evaporation is the primary ablation mechanism with minimal surface disturbance. Conversely, Inconel 718 exhibited increased roughness, particularly at lower scanning speeds and varying fluence levels, where melt expulsion played a significant role. These results highlight the critical role of scanning speed and fluence in optimizing surface quality for different materials, emphasizing the necessity for tailored laser processing settings to achieve desired ablation outcomes. This analysis not only advances our understanding of laser-material interactions but also guides the optimization strategies for ns-pulsed laser applications in industry. The 3D confocal microscopy image of an ablated trench at the optimal surface roughness of both materials is shown in Fig. 11. The ablation conditions that correspond to the optimal (minimum) surface roughness of both materials are 23.74 J/cm^2^, 821 mm/s for MMPCD (*Ra* = 0.23 μm), see Fig. 11a, and 53.63 J/cm^2^, 713 mm/s for Inconel 718 (*Ra* = 0.33 μm) as shown in Fig. 11b.

### Mathematical model vs xperimental results

#### Ablation rate comparison

Using MATLAB regression toolbox, the ablation rates of MMPCD and Inconel 718 are obtained in (4) and (5), respectively. The standard error of the regression (S) and the R-squared (R^2^) values are presented in Table 4 indicating that the regression fit is acceptable as the R^2^ values of both models is greater than 85%.

4 $$\begin{aligned}\:{MRR}_{MMPCD}\:&=\:0.05781-0.00071{V}_{n}+0.00148{F}_{n}-0.01024{{V}_{n}}^{2}-0.00886{{F}_{n}}^{2}-0.00248{V}_{n}{F}_{n}-0.0207{{V}_{n}}^{3}+0.00747{{F}_{n}}^{3}\\ &+0.02198{{V}_{n}}^{2}{F}_{n}-0.0080{V}_{n}{{F}_{n}}^{2}-0.00562{{V}_{n}}^{4}+0.00009{{F}_{n}}^{4}+0.01441{{V}_{n}}^{3}{F}_{n}\\ &+0.00584{{V}_{n}}^{2}{{F}_{n}}^{2}-0.01097{V}_{n}{{F}_{n}}^{3}+0.01936{{V}_{n}}^{5}-0.01464{{V}_{n}}^{4}{F}_{n}\\ &+0.01872{{V}_{n}}^{3}{{F}_{n}}^{2}-0.00883{{V}_{n}}^{2}{{F}_{n}}^{3}-0.00851{V}_{n}{{F}_{n}}^{4}\end{aligned}$$

5 $$\begin{aligned}\:{MRR}_{Inconel\:718}&=0.0346+0.0487{F}_{n}+0.0078{V}_{n}+0.0136{{F}_{n}}^{2}-0.033{{V}_{n}}^{2}\:\\ & +0.0503{F}_{n}{V}_{n}-0.004{{V}_{n}}^{3}+0.0706{{F}_{n}}^{2}{V}_{n}-0.068{F}_{n}{{V}_{n}}^{2}\:+0.1557{{V}_{n}}^{4}-0.0176{{F}_{n}}^{2}{{V}_{n}}^{2}\\ & -0.0699{F}_{n}{{V}_{n}}^{3}-0.092{{V}_{n}}^{5}-0.0745{{F}_{n}}^{2}{{V}_{n}}^{3}+0.087{F}_{n}{{V}_{n}}^{4}\end{aligned}$$

**Fig. 12:**
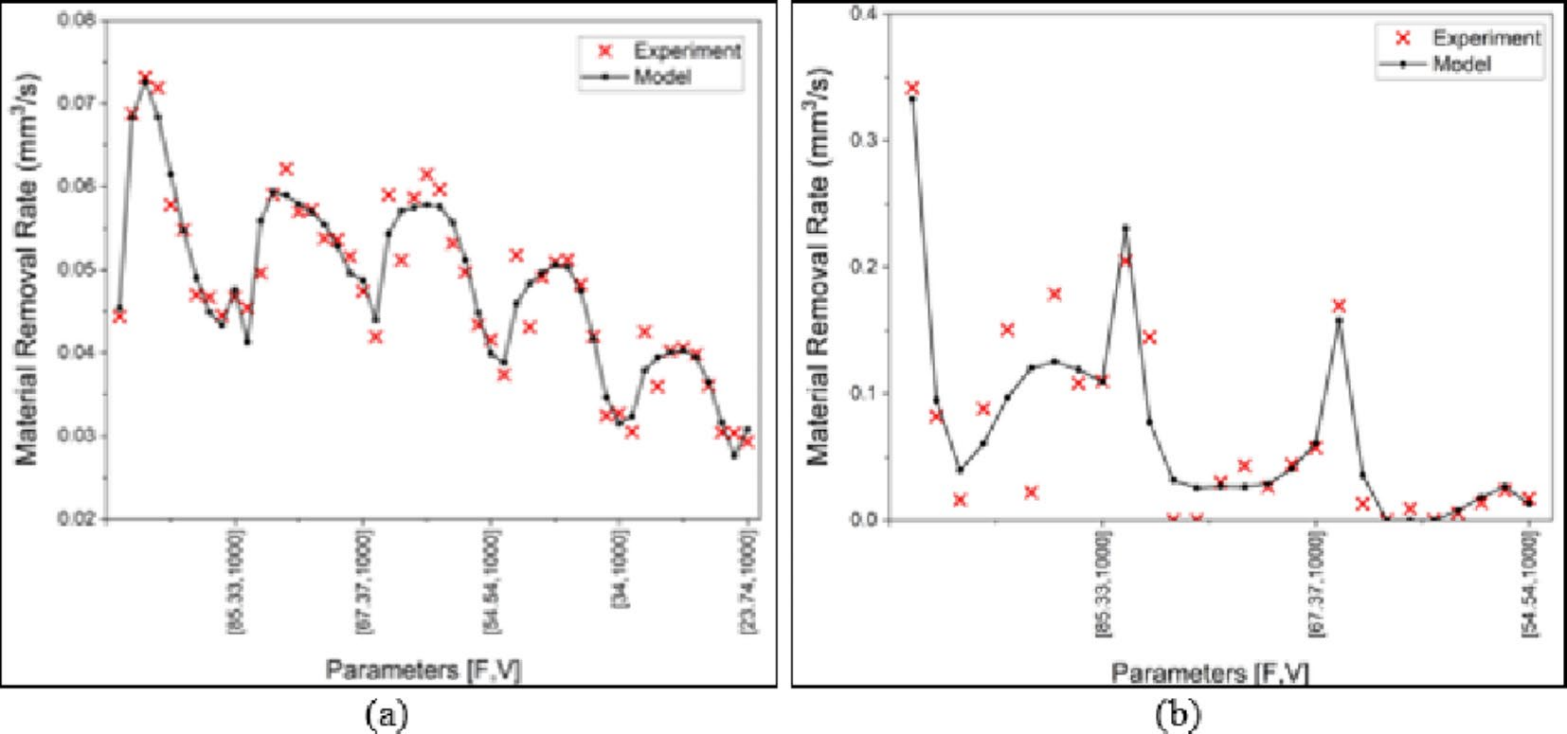
Experiment vs. model ablation rate comparison (a) MMPCD, (b) Inconel 718.

Figure 12 depicts the comparison plot between experimental and regression model results. In Fig. 12a, the good fit between the experiment and model results indicated by the values in Table 4 is obviously visualized. The difference between the R-squared values of both materials is clearly illustrated in Fig. 12b as the fit of the regression model of the Inconel 718 is lower than the MMPCD. However, both mathematical models are acceptable, and they are suitable to be promoted as optimization objective functions.

**Table 4:** Ablation rate mathematical models’ summary.

Item	MMPCD	Inconel 718
Standard Error of the Regression (S)	0.00329	0.0457
R-Squared (R^2^)	94.12 %	85.94 %

#### Surface roughness comparison

Similarly, the surface roughness mathematical models are developed using the MATLAB regression toolbox. The two materials’ models are shown in (6) and (7). However, the MMPCD model used box-cox transformation with λ value − 1. Table 5 presents the regression models fitness indicators values S and R^2^. The latter values indicate goodness of fit as they are above 85%. The S value of the MMPCD model is slightly high value, however, the model is acceptable.

6 $$\begin{aligned}\:-{{Ra}_{MMPCD}}^{-1}&=3.1619-0.966{V}_{n}+0.436{F}_{n}+0.333{{V}_{n}}^{2}+1.21{{F}_{n}}^{2}\\ & +\:0.071{V}_{n}{F}_{n}+2.293{{V}_{n}}^{3}-0.162\:{{F}_{n}}^{3}+0.663{{V}_{n}}^{2}{F}_{n}-0.938{V}_{n}{{F}_{n}}^{2}\\ & -\:0.169{{V}_{n}}^{4}-1.246{{F}_{n}}^{4}+0.097{{V}_{n}}^{3}{F}_{n}-0.100{{V}_{n}}^{2}{{F}_{n}}^{2}+0.242{V}_{n}{{F}_{n}}^{3}-\:1.609{{V}_{n}}^{5}-1.214{{V}_{n}}^{4}{F}_{n}\\ & +0.479{{V}_{n}}^{3}{{F}_{n}}^{2}+0.087{{V}_{n}}^{2}{{F}_{n}}^{3}+0.357{V}_{n}{{F}_{n}}^{4}\end{aligned}$$

7 $$\begin{aligned}\:{Ra}_{Inconel\:718}&=0.6453+0.1636{F}_{n}-0.097{V}_{n}-0.0914{{F}_{n}}^{2}\\ &+0.775{{V}_{n}}^{2}+0.4816{F}_{n}{V}_{n}-0.642{{V}_{n}}^{3}-0.070{{F}_{n}}^{2}{V}_{n}-0.979{F}_{n}{{V}_{n}}^{2}\\ &-0.350{{V}_{n}}^{4}-0.143{{F}_{n}}^{2}{{V}_{n}}^{2}-0.551{F}_{n}{{V}_{n}}^{3}\\ &+0.526{{V}_{n}}^{5}+0.134{{F}_{n}}^{2}{{V}_{n}}^{3}+0.978{F}_{n}{{V}_{n}}^{4}\end{aligned}$$

**Fig. 13:**
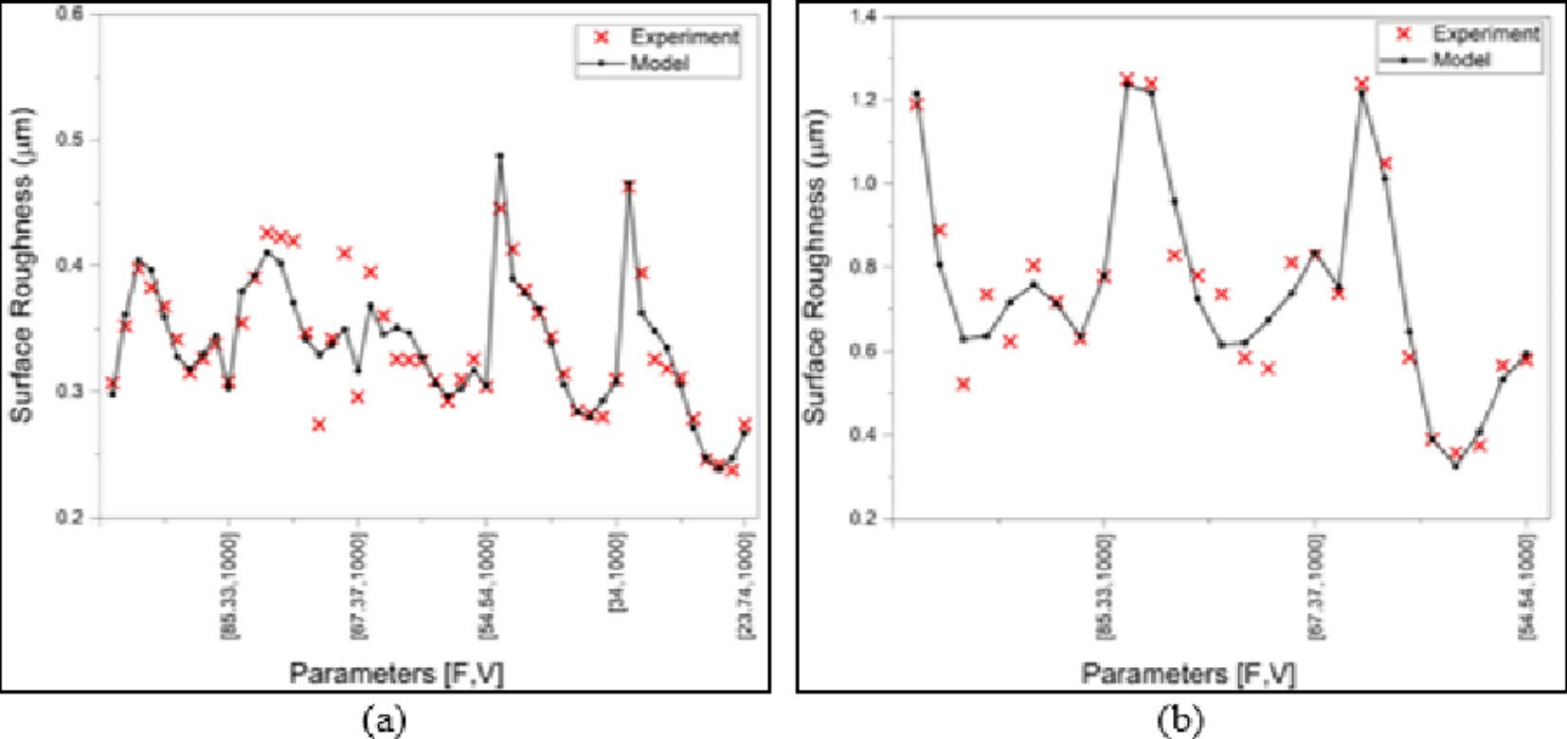
Experiment vs. model surface roughness comparison (**a**) MMPCD, (**b**) Inconel 718.

The visualization of the fitness between the experiment and model results is illustrated in Figure 13. Again, both models can be relied on for the next stage as objectives of the multi-objective optimization.

**Table 5:** Surface roughness mathematical models’ summary.

**Item**	**MMPCD**	**Inconel 718**
**Standard Error of the Regression (S)**	0.2	0.093
R-Squared (R^2^)	89.29 %	93.91 %

### Multi-objective optimization results

#### WVGWO optimization results

**Fig. 14:**
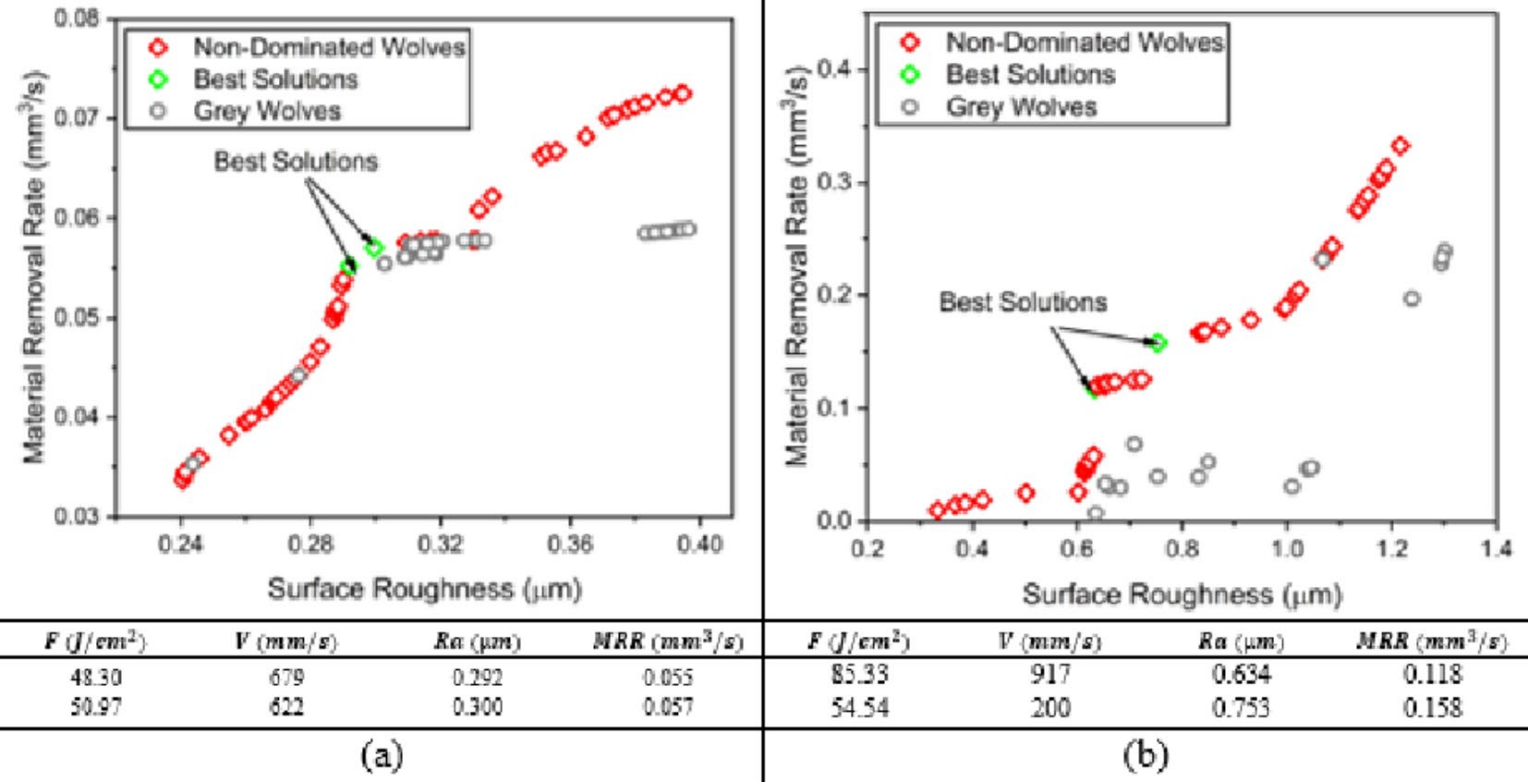
WVGWO pareto front (**a**) MMPCD and (**b**) Inconel 718.

The weighted value gray wolf optimizer simulates the prey hunting of wolves’ pack. A wolf pack has three ranks; alpha wolf, beta wolf and the remaining wolves are called omega wolves. Hence, this algorithm starts with a random population that search for a pray (an optima). A best fitness value with a corresponding solution is given the name non-dominated wolf. Further iterations of hunting, all best fitness values are called non-dominated wolves as shown in Fig. 14, which represents the pareto front as in Fig. 4, where the remaining wolves are called gray wolves. The mathematical model of the prey hunting movement is illustrated in details in^47,48^. The results show that the optimal running conditions of MMPCD material are two approximate equal parameter sets, however, the surface roughness and material removal rates are nearly the same for both solutions; Ra = 0.292 μm and 0.3 μm, MRR = 0.055 and 0.057 mm^3^/s, respectively. Also, the Inconel 718 material has two optimal solutions. At higher fluence and scanning speeds, one can obtain a surface roughness of 0.634 μm and MRR of 0.118 mm^3^/s, if the surface roughness is a priority. Meanwhile, at low-level of fluence and low scanning speed the material removal rate improves while the surface roughness is increased.

#### MOPS optimization results

**Fig. 15:**
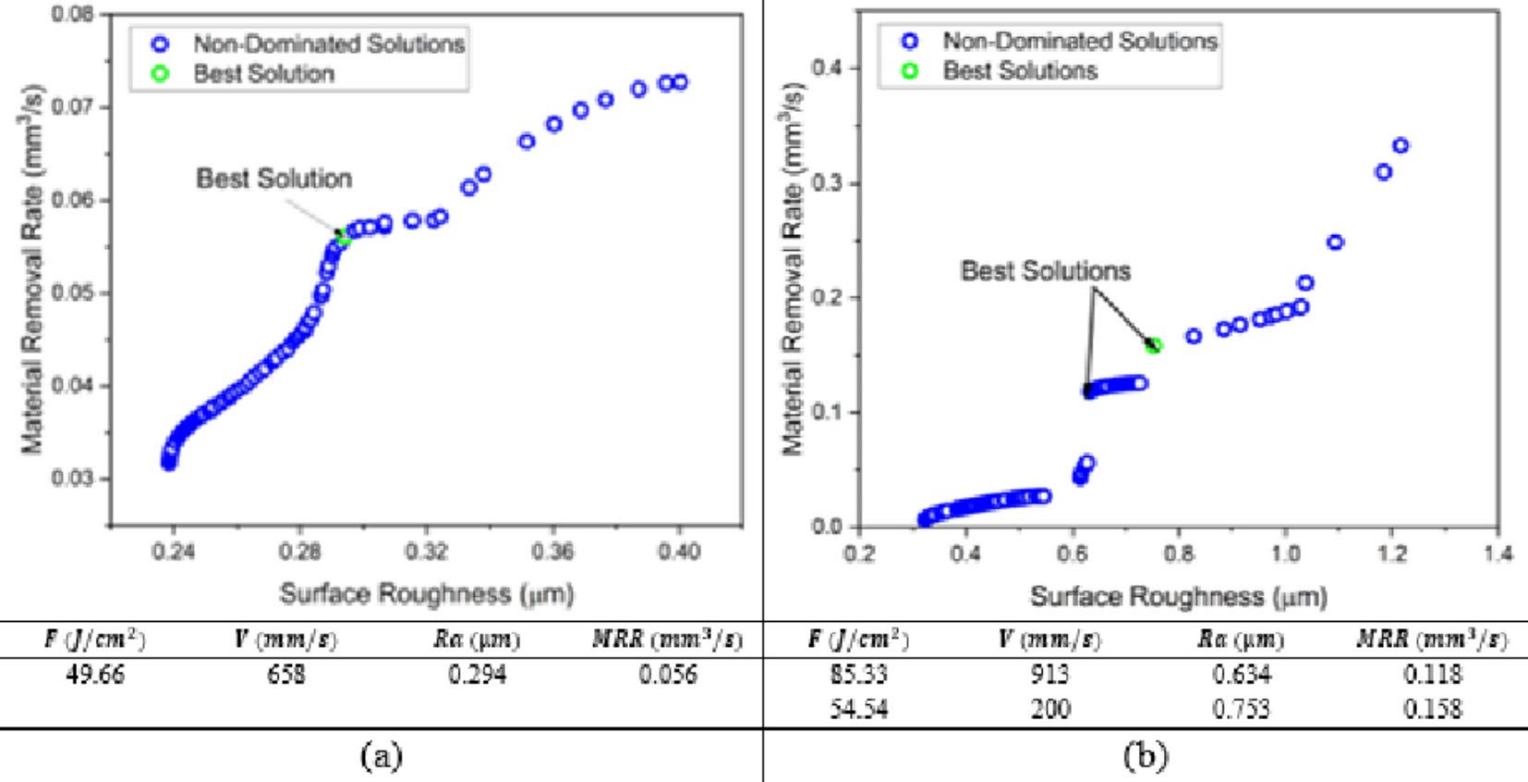
MOPS pareto front (a) MMPCD and (b) Inconel 718.

In Fig. 15, using maximum of iterations 10,000, pareto size 100 and constraint tolerance 10^−10^, the results of the multi-objective pareto search algorithm on MATLAB agrees with the results of WVGWO. For the MMPCD material, MOPS algorithm presents a better optimal solution the entails a slight improvement in the MRR with corresponding fluence of 49.66 J/cm^2^ and scanning speed of 658 mm/s which are likely the average between the two solutions obtained by WVGWO. In case of the Inconel 718, same solutions are obtained.

#### MOGA optimization results

**Fig. 16:**
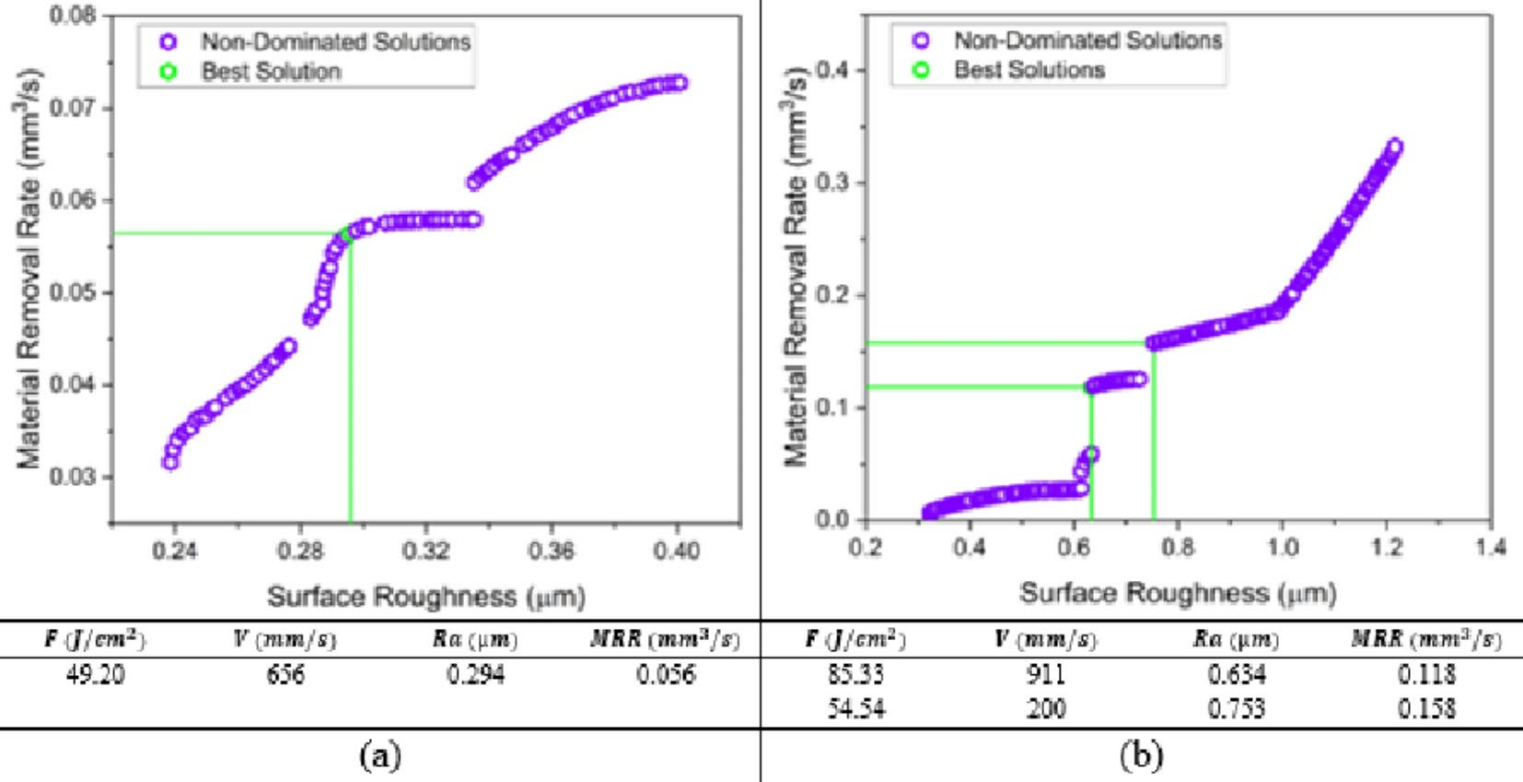
MOGA pareto front (a) MMPCD and (b) Inconel 718.

Additionally, the multi-objective genetic algorithm (MOGA) is used to confirm the previous algorithms. The population size used is 100, maximum generations are 2000, maximum stall generations is 500 and function tolerance of 10^−10^. Surprisingly, the same optimal solutions are achieved as shown in Fig. 16.

#### MOSFO optimization results

**Fig. 17:**
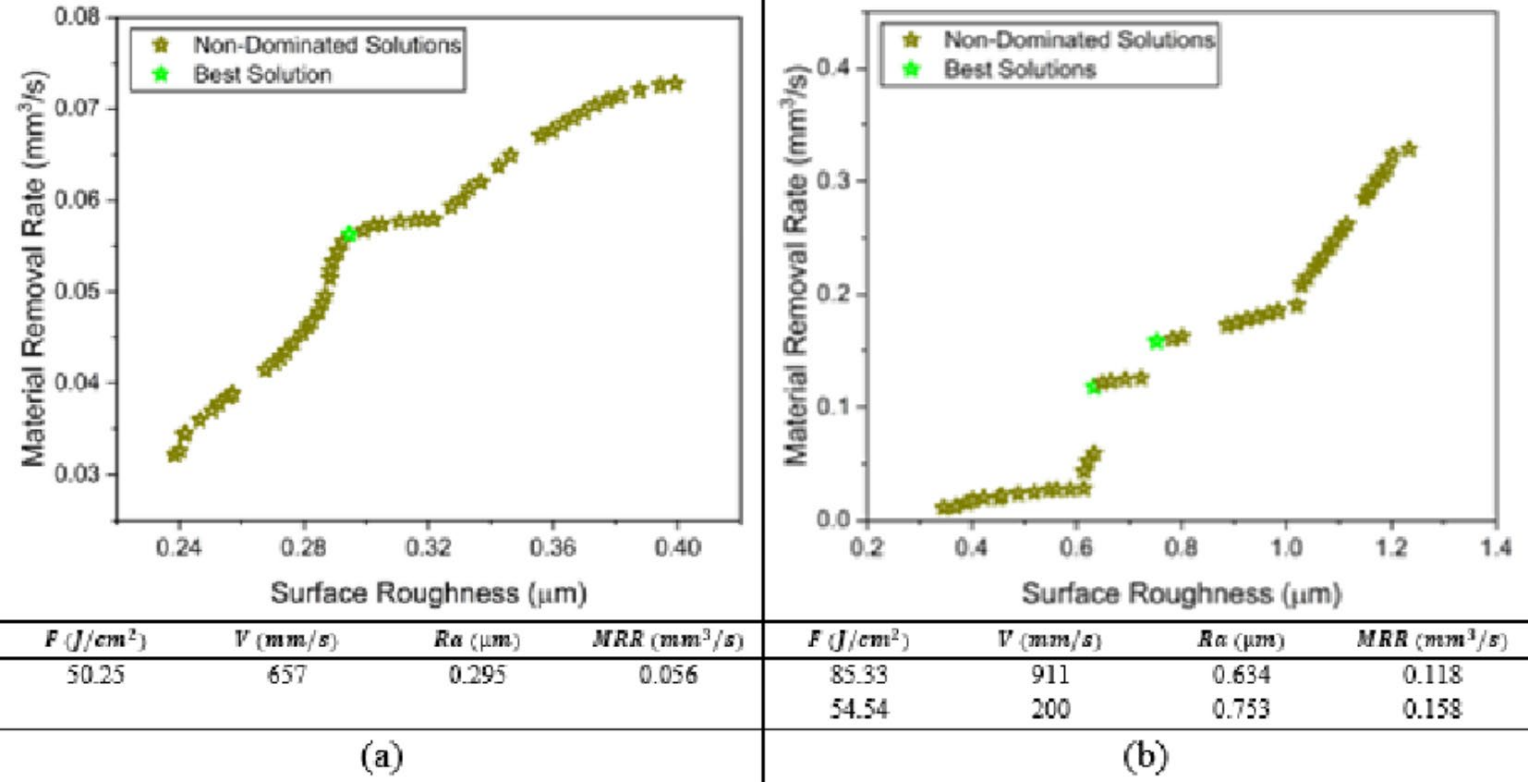
MOSFO pareto front (**a**) MMPCD and (**b**) Inconel 718.

The new novel multi-objective sunflower optimization is invited to this research in order to double confirm the optimal running conditions of the laser ablation of MMPCD and Inconel 718. Undoubtedly, it seems that all algorithms agree on certain optimal solutions for both materials. In Fig. 17, the optimal running conditions of MMPCD are fluence 50.25 J/cm^2^ and scanning speed of 657 mm/s that achieve a surface roughness of 0.295 μm and MRR of 0.056 mm^3^/s, which offers no much improvement compared to the other used algorithms. Meanwhile, the same two optimal running conditions of Inconel 718 are obtained as shown in Fig. 17b.

To wrap up, Table 6 summarizes the multi-objective optimization carried out in this research in a comparison way. The results show that MMPCD model has one optimal solution obtained from all used algorithms. While the Inconel 718 material has two optimal solutions on the pareto front representing a trade-off choice between the surface roughness and material removal rate.

**Table 6:** Optimization results summary.

Material	Best	$\:\varvec{F}\:(\varvec{J}/{\varvec{c}\varvec{m}}^{2})$	$\:\varvec{V}\:(\varvec{m}\varvec{m}/\varvec{s})$	Ra (µm)	MRR (mm^3^/s)
MMPCD	MOGA MOPS	49.2 49.66	656 658	0.294	0.056
Inconel 718	All	85.33	911, 913, 917	0.634	0.118
		54.54	200	0.753	0.158

On comparing the optimization results with the experimental results, the obtain results of Inconel 718 already exist in the experimented trials. While the optimal results of MMPCD material are compared to the nearest experimental trail as shown in Table 7.

**Table 7:** Validation of the optimal results compared to the experimental results.

Material	Optimal				Measured				Rel. Err. Ra	Rel. Err. MRR
	F	V	Ra	MRR	F	V	Ra	MRR		
MMPCD	49.66	658	0.294	0.056	54.54	700	0.293	0.053	+ 0.34%	+ 5.66%
Inconel 718	85.33	913	0.634	0.118	85.33	900	0.63	0.108	+ 0.63%	+ 9.26% ^1^
	54.54	200	0.753	0.158	54.54	200	0.738	0.17	+ 2.03	−7.06% ^2^

^1^ Optimal result 1.

^2^ Optimal result 2.

**Fig. 18:**
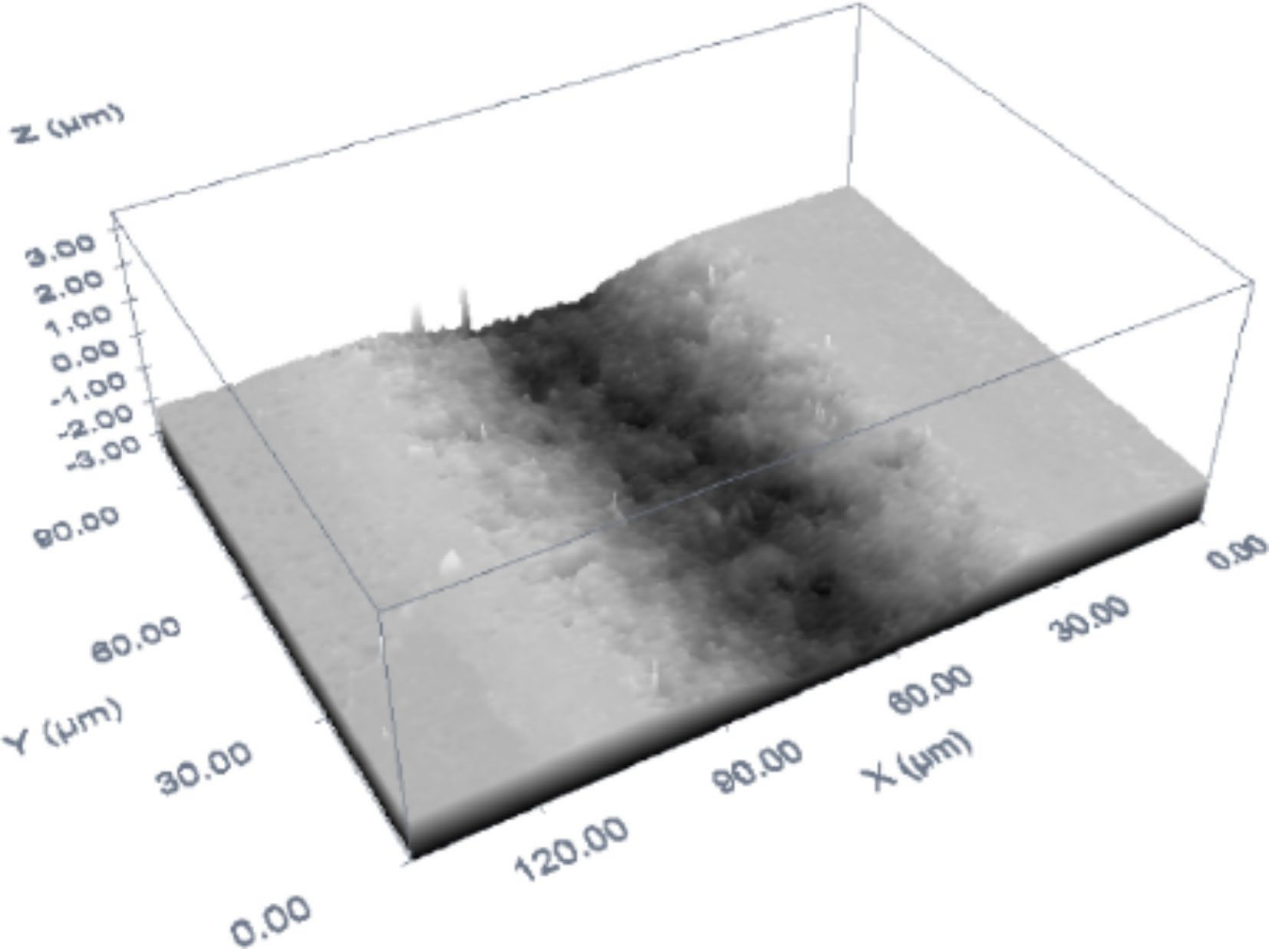
3D confocal microscopic image of ablated trench of optimal ablation rate and surface roughness results of MMPCD.

**Fig. 19:**
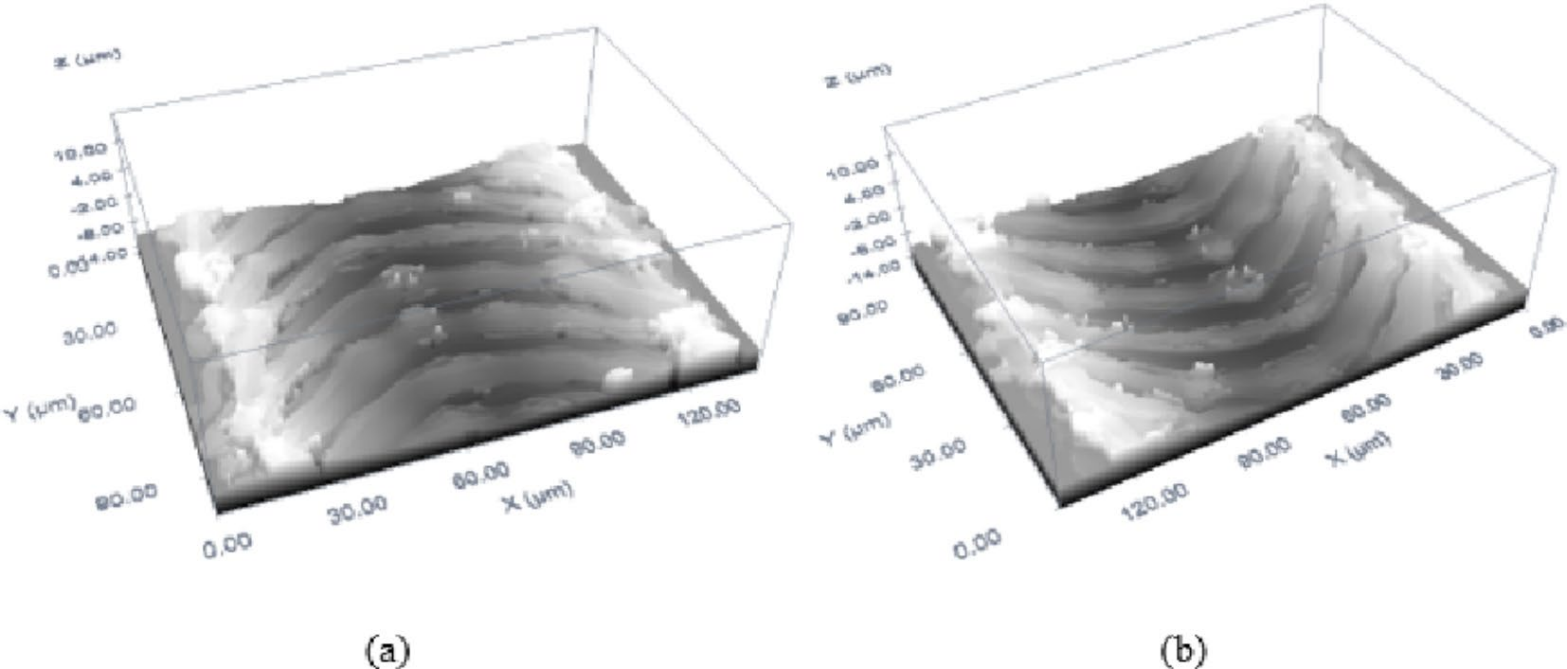
3D confocal microscopic image of ablated trench of optimal ablation rate and surface roughness results of Inconel 718 (**a**) Case 1 and (**b**) Case 2.

Finally, the samples of optimal ablation conditions stated in Table 7 are captured to produce a 3D confocal microscopic image for the only case of MMPCD in Fig. 18, and the two optimal cases of Inconel 718 in Fig. 19.

### Conclusion

This paper has presented an experimental investigation into the effect of laser fluence and scanning speed on the obtainable ablation rate and resultant surface roughness of MMPCD and Inconel 718 when ablated using a ns-pulsed laser. Mathematical regression models have been developed based on the experimental results for the effective ablation rate and surface roughness for the range of fluence and scanning speed investigated. Four multi-objective optimization algorithms: WVGWO, MOPS, MOGA, and MOSFO, were used to optimize the regression models to determine the optimal process conditions and best possible performance when ablating the given materials. Optimum conditions were identified for maximum effective ablation rates and minimum surface roughness and they were experimentally validated. The results generally showed good agreement between the predicted values and the experimental results. The findings of this work are as follows:

- MMPCD and Inconel 718 demonstrated distinctly different responses to the laser ablation process. For MMPCD, the lowest surface roughness measured was 0.24 μm at a fluence of 23.73 J/cm² and scanning speeds between 700 and 950 mm/s, indicating high thermal conductivity’s role in favorable outcomes. Conversely, Inconel 718 exhibited higher surface roughness, up to 1.35 μm, particularly at a fluence of more than 60 J/cm² and scanning speeds between 200 and 300 mm/s, demonstrating its susceptibility to thermal effects and melt expulsion.
- The experimental results quantified the impact of fluence and scanning speed on ablation efficiency. Fluence was the dominant factor in determining ablation rate, particularly noted with Inconel 718 where the ablation rate decreased significantly at lower fluences (less than 54.54 J/cm²). Scanning speed had a more pronounced effect on surface roughness, where higher speeds typically resulted in smoother surfaces.
- MMPCD showed a consistent response across various input ranges, maintaining surface roughness well within the optimal threshold. In contrast, Inconel 718 displayed no effective ablation rate at certain fluence and scanning speed ranges, emphasizing the need for precise parameter control.
- The ablation mechanisms were heavily influenced by the materials’ properties. MMPCD primarily underwent evaporation, maintaining structural integrity and resulting in cleaner surfaces. In contrast, Inconel 718 experienced significant melt expulsion, which often adversely affected both ablation rate and surface quality.
- Although no single multi-objective optimization algorithm outperformed others, all algorithms consistently identified similar optimal settings for each material, validating our experimental design. This is due to the fact that the global minima of the ns-pulsed laser machining in the selected parameter region is captured by all algorithms. This conclusion suggests a future concern of investigating wider range of ablation conditions in order to find more minima and competitiveness of algorithms.
- For MMPCD, optimal results were achieved at a medium level of fluence and scanning speed. For Inconel 718, higher fluence and scanning speeds (above 60 J/cm² and 200–300 mm/s, respectively) were optimal for achieving better surface roughness, whereas lower settings (fluence of 54.54 J/cm² and scanning speed of 200 mm/s) optimized the ablation rate.

The results presented here offer a reliable means to identify the processing window and ablation conditions for optimal performance of ns-pulsed laser ablation when processing MMPCD and Inconel 718. Further investigation of the effect of the examined parameters on microstructure modifications will be carried out in future work. The findings of this research study will be used to ablate 3D shapes using overlapped trenches. The next step will be to optimize the overlap distance to produce the highest ablation rate while minimizing surface roughness.

## Data Availability

The datasets used and/or analysed during the current study available from the corresponding author on reasonable request.
